# PARP1 Deficiency Attenuates Cisplatin Nephrotoxicity and Renal COX-2/PGE2 Response

**DOI:** 10.3390/cells15191810

**Published:** 2026-10-03

**Authors:** Theophilus Bhatti, Jia-Bin Yu, Ji-Yeong Bae, Jinu Kim

**Affiliations:** 1 Interdisciplinary Graduate Program in Advanced Convergence Technology & Science, Jeju National University, Jeju 63243, Republic of Korea; theophilus@stu.jejunu.ac.kr (T.B.); jybae@jejunu.ac.kr (J.-Y.B.); 2 Department of Anatomy, College of Medicine, Jeju National University, Jeju 63243, Republic of Korea; yujiabin1993@jejunu.ac.kr; 3 College of Pharmacy, Jeju National University, Jeju 63243, Republic of Korea

**Keywords:** cisplatin, acute kidney injury, nephrotoxicity, PARP1, *Ptgs2*, cyclooxygenase-2, prostaglandin E2, inflammation, tubular injury, chemokine

## Abstract

**Highlights:**

**What are the main findings?**

- PARP1 deficiency attenuates cisplatin-induced renal dysfunction, tubular injury, and inflammatory gene expression.
- Loss of PARP1 markedly suppresses the cisplatin-induced renal *Ptgs2*/COX-2/PGE2 response.

**What are the implications of the main findings?**

- The renal COX-2/PGE2 response represents a potential downstream molecular response associated with PARP1 during cisplatin nephrotoxicity.
- The findings support a potential PARP1–NF-κB–COX-2/PGE2 signaling axis associated with cisplatin-induced kidney inflammation.

**Abstract:**

Cisplatin is a widely used chemotherapeutic agent whose clinical utility is limited by nephrotoxicity, characterized by tubular injury and renal inflammation. Poly(ADP-ribose) polymerase 1 (PARP1) contributes to cisplatin-induced kidney injury and inflammatory signaling, but the downstream inflammatory pathways involved remain incompletely defined. Here, we investigated whether PARP1 modulates the renal cyclooxygenase-2 (COX-2)/prostaglandin E2 (PGE2) response during cisplatin-induced acute kidney injury (AKI). Female wild-type and *Parp1*-knockout (KO) mice on a C57BL/6 background received a single intraperitoneal injection of cisplatin (20 mg/kg) and were evaluated at days 1 and 4, while male mice were evaluated at day 4 for renal function. Renal function, tubular injury, inflammatory gene expression, NF-κB activation, *Ptgs2* mRNA and COX-2 protein expression, and kidney PGE2 levels were assessed. Cisplatin markedly increased plasma creatinine and blood urea nitrogen levels and induced tubular injury in both the renal cortex and outer stripe of the outer medulla, whereas these changes were significantly attenuated in *Parp1* KO mice at day 4. At day 4, cisplatin-induced renal expression of *Il1b*, *Il6*, *Ccl5*, *Cxcl1*, and *Cxcl2* was also substantially reduced by *Parp1* deficiency. Cisplatin increased renal NF-κB p65 activation, as assessed by the p-p65/t-p65 ratio, in wild-type mice, whereas this response was significantly attenuated in *Parp1* KO mice. Consistently, cisplatin increased renal *Ptgs2* mRNA expression, COX-2 protein abundance, and PGE2 levels in wild-type mice, whereas all three responses were significantly attenuated in *Parp1* KO mice. In particular, the cisplatin-induced increase in renal PGE2 was nearly abolished in the absence of PARP1. These findings demonstrate that PARP1 deficiency attenuates cisplatin-induced renal dysfunction, tubular injury, and inflammatory responses and identify suppression of NF-κB activation and the COX-2/PGE2 response as features of this protective phenotype. The results support a potential PARP1–NF-κB–COX-2/PGE2 signaling axis in cisplatin nephrotoxicity.

## 1. Introduction

Cisplatin remains one of the most widely used chemotherapeutic agents for the treatment of solid tumors, but its clinical utility is constrained by dose-limiting nephrotoxicity, which develops in a substantial proportion of treated patients [1,2,3]. Following systemic administration, cisplatin is preferentially taken up by proximal renal tubular epithelial cells through basolateral membrane transporters, including organic cation transporter 2 and copper transporter 1, resulting in renal accumulation of cisplatin at concentrations higher than those in the circulation [4,5,6]. This preferential renal accumulation renders the proximal tubule particularly susceptible to cisplatin-induced injury and contributes to the development of acute kidney injury (AKI) during cisplatin-based chemotherapy [7,8,9].

Beyond direct tubular cytotoxicity, accumulating evidence indicates that innate inflammatory responses have a critical role in the development and progression of cisplatin-induced AKI [10,11,12]. Cisplatin-induced tubular cell injury results in the release of damage-associated molecular patterns [13,14,15]. These molecules activate resident and infiltrating immune cells and trigger the production of pro-inflammatory cytokines and chemokines, including *Il1b*, *Il6*, *Ccl5*, *Cxcl1*, and *Cxcl2* [16,17,18]. This inflammatory response is mediated in part through toll-like receptor 4 (TLR4)/nuclear factor-κB (NF-κB) signaling and further amplifies tubular injury, establishing a feed-forward cycle of tissue damage and inflammation [19,20,21].

Poly(ADP-ribose) polymerase 1 (PARP1) is a nuclear enzyme best known for its role in DNA damage recognition and repair, where it catalyzes the transfer of ADP-ribose units from nicotinamide adenine dinucleotide (NAD^+^) to itself and other chromatin-associated proteins [22,23,24]. Excessive PARP1 activation, however, extends beyond its physiological role in DNA repair and can cause cellular NAD^+^ and adenosine triphosphate (ATP) depletion, thereby contributing to energy failure and cell death [25,26,27]. PARP1 also participates in inflammatory transcriptional regulation, including DNA damage-induced NF-κB activation [28,29,30]. In cisplatin nephrotoxicity, PARP1 activation has been implicated in renal dysfunction, tubular injury, oxidative stress, and inflammatory signaling, whereas genetic deletion or pharmacological inhibition of PARP1 attenuates these responses in experimental models [31,32,33]. However, the specific downstream inflammatory effector pathways through which PARP1 amplifies the renal inflammatory response to cisplatin remain incompletely defined.

Cyclooxygenase-2 (COX-2), encoded by *Ptgs2*, is a highly regulated cyclooxygenase isoform that catalyzes the conversion of arachidonic acid to prostanoid precursors, with prostaglandin E2 (PGE2) serving as an important mediator of renal inflammatory and pathophysiological responses [34,35,36]. In cisplatin-induced nephrotoxicity, renal COX-2 expression and PGE_2_ production have been reported to increase, implicating the COX-2/PGE2 pathway in the development of renal injury and inflammation [37,38,39]. Because PARP1 can potentiate NF-κB-dependent transcriptional responses [40,41,42], PARP1 represents a plausible upstream regulator of the renal COX-2/PGE2 response to cisplatin. However, whether PARP1 contributes to activation of the COX-2/PGE2 pathway during cisplatin nephrotoxicity remains unclear. Although the renoprotective and anti-inflammatory effects of PARP1 deficiency have been described previously, the relationship between PARP1 and the renal COX-2/PGE2 response has not been defined. Therefore, in the present study, we investigated whether PARP1 deficiency modulates the COX-2/PGE2 pathway during cisplatin-induced kidney injury. We hypothesized that loss of PARP1 attenuates cisplatin-induced renal inflammation in association with suppression of the COX-2/PGE2 response.

## 2. Materials and Methods

### 2.1. Animals and Experimental Design

Eight- to ten-week-old female and male wild-type and *Parp1*-knockout (KO) mice on a C57BL/6 background were used in this study. The *Parp1* KO strain was originally obtained from Jackson Laboratory (Bar Harbor, ME, USA; no. 002779) on a 129S background and was backcrossed with C57BL/6 wild-type mice for 11 generations before establishing the homozygous C57BL/6 *Parp1* KO line used in this study [43]. Age-matched C57BL/6 wild-type mice served as controls. The animals had not undergone any previous experimental procedures before enrollment in this study. Mice were housed under specific pathogen-free conditions with controlled temperature and humidity and a 12-h light/12-h dark cycle, with free access to standard laboratory chow and water. Environmental enrichment, including nesting material, was provided throughout the housing period. Mice were acclimatized to the animal facility for 7 days before the experiments. Animals were monitored daily for general health and signs of pain or distress, and all procedures were performed with appropriate handling to minimize unnecessary suffering. Humane endpoints were established in accordance with the approved Institutional Animal Care and Use Committee (IACUC) protocol and included a loss of 20% or more of initial body weight. No unexpected adverse events requiring intervention or early termination were observed during the experimental period. All animal experiments were designed and reported in accordance with the ARRIVE guidelines [44]. All animal procedures were approved by the IACUC of Jeju National University (approval no. 2023-0038). Mice were assigned to the experimental groups using a simple randomization procedure based on randomly generated numbers. The four groups were wild-type control, *Parp1* KO control, wild-type cisplatin, and *Parp1* KO cisplatin. To minimize potential confounding, the order of treatments and outcome measurements was varied across experimental groups, and animals from different groups were distributed across cage locations. Investigators were aware of group allocation during animal allocation and experimental procedures. Histological outcome assessment was performed in a blinded manner, whereas molecular, biochemical, and statistical analyses were not blinded to group allocation. For each experimental cohort, 8 mice were allocated to each of the four experimental groups. Female mice were evaluated at days 1 and 4; renal function and quantitative real-time polymerase chain reaction (qRT-PCR) analyses were performed at both time points, whereas histological, Western blot, and PGE2 analyses were performed at day 4. Male mice were evaluated at day 4 for renal function only. The experimental unit was an individual mouse. The sample size was determined based on our previous studies using the same cisplatin-induced kidney injury model [25,32,45]; no formal a priori sample size calculation was performed. No a priori inclusion or exclusion criteria were established for animals or data points. No animals or data points were excluded after group allocation. For Western blot analysis at day 4, 6 female mice per group were randomly selected from the 8 mice allocated to each group. Cisplatin-induced kidney injury was induced as previously described [32,45]. Cisplatin (Sigma-Aldrich, St. Louis, MO, USA; no. P4394) was administered as a single intraperitoneal injection at 20 mg/kg body weight, whereas control mice received an equivalent volume of vehicle. Female mice were euthanized 1 or 4 days after treatment, whereas male mice were euthanized 4 days after treatment. Mice were deeply anesthetized with pentobarbital sodium (120 mg/kg body weight, intraperitoneally; Hanlim Pharm, Seoul, Republic of Korea). Blood was collected under deep anesthesia, and the mice were euthanized by exsanguination before kidney harvest. Kidney tissues were either fixed in 4% paraformaldehyde (T&I, Chuncheon, Gangwon, Republic of Korea; no. BPP-9004) for histological analysis or snap-frozen and stored at −80 °C until use.

### 2.2. Assessment of Renal Function

Blood samples were collected under deep anesthesia during the terminal procedure and centrifuged to obtain plasma. Plasma creatinine and blood urea nitrogen (BUN) concentrations were measured as previously described [46,47], using a creatinine assay kit (BIOMAX, Guri, Gyeonggi, Republic of Korea; no. BM-CRE-100) and a urea nitrogen assay kit (Thermo Fisher Scientific, Cleveland, OH, USA; no. EIABUN), respectively, according to the manufacturers’ instructions. Concentrations were calculated from standard curves and expressed as mg/dL.

### 2.3. Histological Analysis and Tubular Injury Scoring

Kidney tissues were fixed in 4% paraformaldehyde, embedded in paraffin, sectioned, and stained with a periodic acid–Schiff (PAS) stain kit (Abcam, Cambridge, UK; no. ab245886). Tubular injury was evaluated separately in the renal cortex and outer stripe of the outer medulla (OSOM), as previously described [48,49]. Tubules exhibiting tubular dilation, cast formation, epithelial cell sloughing, and/or loss of the brush border were considered injured. Five randomly selected high-power fields per kidney were evaluated in a blinded manner. The tubular injury score was calculated as the percentage of injured tubules relative to the total number of tubules examined.

### 2.4. qRT-PCR

Total RNA was extracted from kidney tissues using TRIzol reagent (Thermo Fisher Scientific; no. 15596026) according to the manufacturer’s instructions. Complementary DNA was synthesized from total RNA using the LaboPass cDNA synthesis kit (Cosmogenetech, Seoul, Republic of Korea; no. CMRTK001). qRT-PCR was performed using a CFX connect real-time PCR detection system (Bio-Rad Laboratories, Hercules, CA, USA; no. 185-5201) and KAPA SYBR FAST qPCR Master Mix for Bio-Rad iCycler (Kapa Biosystems, Wilmington, MA, USA; no. KK4608). Relative mRNA expression was calculated using the 2^−ΔΔCt^ method and normalized to *Ppia*, as previously described [50,51]. Expression levels were expressed relative to those in the corresponding wild-type control group. The genes analyzed were *Il1b*, *Il6*, *Ccl5*, *Cxcl1*, *Cxcl2*, and *Ptgs2*. The primer sequences were as follows: *Il1b*, forward 5′-CTCCATGAGCTTTGTACAAGG-3′ and reverse 5′-TGCTGATGTACCAGTTGGGG-3′; *Il6*, forward 5′-TACCACTTCACAAGTCGGAGGC-3′ and reverse 5′-CTGCAAGTGCATCATCGTTGTTC-3′; *Ccl5*, forward 5′-ATATGGCTCGGACACCACTC-3′ and reverse 5′-GTGACAAACACGACTGCAAGA-3′; *Cxcl1*, forward 5′-GCTGGGATTCACCTCAAGAA-3′ and reverse 5′-TGGGGACACCTTTTAGCATC-3′; *Cxcl2*, forward 5′-CATCCAGAGCTTGAGTGTGACG-3′ and reverse 5′-GGCTTCAGGGTCAAGGCAAACT-3′; *Ptgs2*, forward 5′-TGAGTGGGGTGATGAGCAAC-3′ and reverse 5′-AAGTGGTAACCGCTCAGGTG-3′; and *Ppia*, forward 5′-GGCAAATGCTGGACCCAACACA-3′ and reverse 5′-TGCTGGTCTTGCCATTCCTGGA-3′.

### 2.5. Western Blot Analysis

Kidney tissues were homogenized in ice-cold lysis buffer (Thermo Fisher Scientific; no. 78510) supplemented with 1% protease inhibitor cocktail set III (Sigma-Aldrich; no. 539134) and centrifuged at 17,000× *g* for 20 min at 4 °C. Supernatants were collected, and protein concentrations were determined using the Bradford assay (Bio-Rad Laboratories; no. 5000006). Equal amounts of protein (20–40 μg) were separated by SDS–polyacrylamide gel electrophoresis, transferred to polyvinylidene fluoride membranes, and incubated overnight at 4 °C with primary antibodies against phosphorylated NF-κB p65 (p-p65; ABclonal Technology, Woburn, MA, USA; no. AP0123; 1:2500 dilution), total NF-κB p65 (t-p65; ABclonal Technology; no. A22331; 1:2500 dilution), COX-2 (ABclonal Technology; no. A3560; 1:2500 dilution), and β-actin (Santa Cruz Biotechnology, Santa Cruz, CA, USA; no. sc-47778; 1:5000 dilution). After incubation with peroxidase-conjugated anti-rabbit IgG antibody (Vector Laboratories, Newark, CA, USA; no. WB-1000; 1:5000 dilution) or peroxidase-conjugated anti-mouse IgG antibody (Vector Laboratories; no. WB-2000; 1:5000 dilution), protein bands were visualized using a chemiluminescence reagent (PerkinElmer, Boston, MA, USA; no. NEL101) and detected with an Azure c300 imaging system (Azure Biosystems, Dublin, CA, USA). Band intensities were quantified using AzureSpot software (version 1.5.1105; Azure Biosystems). NF-κB p65 activation was expressed as the ratio of p-p65 to t-p65, and COX-2 expression was normalized to β-actin. Western blot procedures and quantification were performed as previously described [52,53].

### 2.6. Measurement of Kidney PGE2

Kidney PGE2 levels were measured using a Prostaglandin E2 express ELISA kit (Cayman Chemical, Ann Arbor, MI, USA; no. 500141) according to the manufacturer’s instructions, as previously applied to the assessment of renal PGE2 in experimental kidney injury [53,54]. Kidney tissues were homogenized in ice-cold homogenization buffer containing 10 mM phosphate (pH 7.4) and 10 μM indomethacin at a ratio of 1 mL buffer per 250 mg tissue. The homogenates were centrifuged at 8000× *g* for 10 min, and the supernatants were collected for analysis. An aliquot of each supernatant was used to determine total protein concentration. For the ELISA, tissue lysates and PGE2 standards were incubated with PGE2-acetylcholinesterase tracer and PGE2 monoclonal antibody in goat anti-mouse IgG-coated plates for 60 min at room temperature on an orbital shaker. After washing, Ellman’s reagent was added, and the plates were developed according to the manufacturer’s instructions. Absorbance was measured at 412 nm using a SpectraMax i3x multi-mode microplate reader (Molecular Devices, San Jose, CA, USA) at the Bio-Health Materials Core-Facility, Jeju National University. PGE2 concentrations were calculated from the standard curve and normalized to the total protein content of each kidney homogenate. Results are expressed as pg/mg protein.

### 2.7. Statistical Analysis

Numeric data were analyzed using SigmaPlot 14.0 (Systat Software, San Jose, CA, USA) and R version 4.5.0 (R Foundation for Statistical Computing, Vienna, Austria), as previously described [54]. Data are presented as the mean ± standard error of the mean (SEM). Normality was assessed using the Shapiro–Wilk test, and homogeneity of variances was evaluated using the Brown–Forsythe test. When required, data were transformed before statistical analysis to better satisfy model assumptions. Logarithmic transformation was applied to plasma creatinine, BUN, and selected mRNA expression data, whereas square-root transformation was used for tubular injury score data. For datasets that satisfied the equal variance assumption, statistical significance was determined using two-way analysis of variance (ANOVA), with cisplatin treatment and genotype as independent factors, followed by the Holm–Sidak multiple-comparison test. When the equal variance assumption was violated, data were analyzed using a two-factor linear model with heteroscedasticity-consistent HC3 robust standard errors [55], and Type III Wald *F* tests were used to evaluate the main effects and interaction. Pairwise comparisons from robust models were adjusted using the Holm method. Data from different time points and sexes were analyzed separately. For *F* statistics reported as *F*_α,β_ = γ, including those from ANOVA and Type III Wald *F* tests, α and β denote the numerator and denominator degrees of freedom, respectively, and γ denotes the *F* value. A *p* value < 0.05 was considered statistically significant. All raw numeric data and corresponding statistical outcomes are provided in the Supplementary Materials.

## 3. Results

### 3.1. Loss of PARP1 Attenuates Cisplatin-Induced Kidney Dysfunction and Tubular Injury

**Figure 1:**
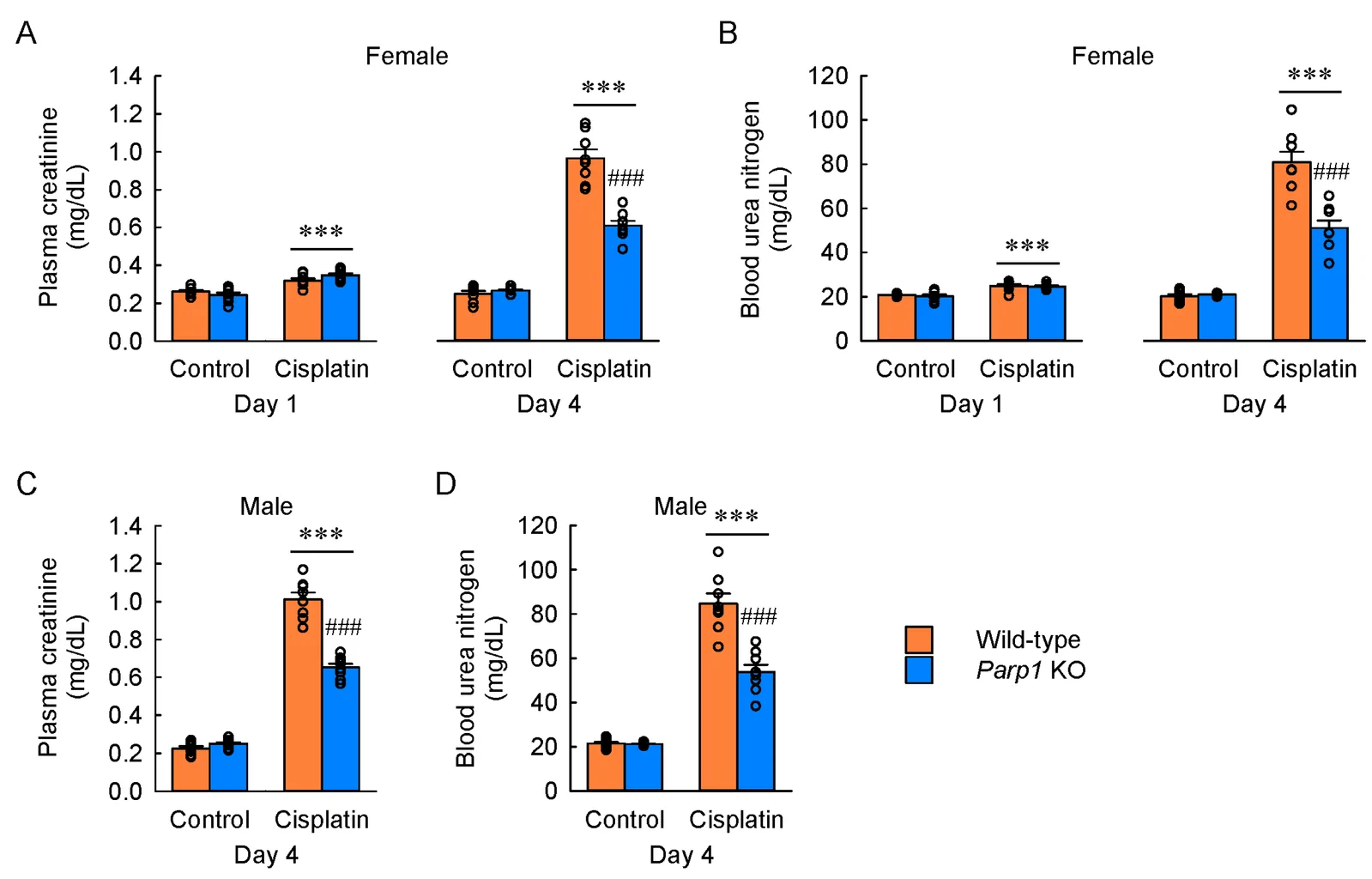
Loss of PARP1 attenuates cisplatin-induced kidney dysfunction. (**A**) Plasma creatinine and (**B**) blood urea nitrogen levels were measured in female wild-type and *Parp1*-knockout (KO) mice at days 1 and 4 after cisplatin or control treatment. (**C**) Plasma creatinine and (**D**) blood urea nitrogen levels were measured in male mice 4 days after treatment. Data are presented as the mean ± SEM (*n* = 8 per group). Female plasma creatinine and blood urea nitrogen at day 1 were analyzed without transformation, whereas the remaining datasets were logarithmically transformed before statistical analysis. Female blood urea nitrogen at day 1 and male blood urea nitrogen at day 4 were analyzed using a two-factor linear model with HC3 robust standard errors and Type III Wald *F* tests, followed by Holm-adjusted pairwise comparisons. The remaining datasets were analyzed using two-way ANOVA followed by the Holm–Sidak post hoc test. *** *p* < 0.001 versus respective control; ### *p* < 0.001 versus respective wild-type.

To determine the effect of PARP1 deficiency on cisplatin-induced kidney injury, renal function was assessed in wild-type and *Parp1* KO mice 1 and 4 days after cisplatin administration in females and 4 days after cisplatin administration in males (Figure 1A–D). At day 1 in female mice, cisplatin significantly increased plasma creatinine levels in both wild-type and *Parp1* KO mice, whereas there was no significant difference between the two genotypes after cisplatin treatment (Figure 1A). Two-way ANOVA revealed a significant main effect of cisplatin (*F*_1,28_ = 56.796, *p* < 0.001), but not of *Parp1* deficiency (*F*_1,28_ = 0.246, *p* = 0.624), together with a significant cisplatin × genotype interaction (*F*_1,28_ = 4.941, *p* = 0.034). Similarly, blood urea nitrogen levels were significantly increased by cisplatin in both genotypes at day 1, with no significant genotype difference after cisplatin treatment (Figure 1B). The two-factor linear model with HC3 robust standard errors showed a significant main effect of cisplatin (*F*_1,28_ = 42.460, *p* < 0.001), but no significant effect of *Parp1* deficiency (*F*_1,28_ = 0.359, *p* = 0.554) or cisplatin × genotype interaction (*F*_1,28_ = 0.048, *p* = 0.828). At day 4 in female mice, cisplatin markedly increased plasma creatinine levels in wild-type mice from 0.25 ± 0.01 to 0.96 ± 0.05 mg/dL (Figure 1A). This increase was significantly attenuated in *Parp1* KO mice, in which plasma creatinine reached 0.61 ± 0.03 mg/dL after cisplatin administration. Baseline plasma creatinine levels were comparable between the two genotypes. Two-way ANOVA of logarithmically transformed data revealed significant main effects of cisplatin (*F*_1,28_ = 538.915, *p* < 0.001) and *Parp1* deficiency (*F*_1,28_ = 16.089, *p* < 0.001), as well as a significant cisplatin × genotype interaction (*F*_1,28_ = 33.109, *p* < 0.001). Similarly, cisplatin increased BUN levels from 20.20 ± 0.86 to 80.94 ± 4.76 mg/dL in wild-type mice (Figure 1B), whereas this increase was significantly attenuated to 50.98 ± 3.47 mg/dL in *Parp1* KO mice. Baseline BUN levels were comparable between the two genotypes. Two-way ANOVA of logarithmically transformed data showed significant main effects of cisplatin (*F*_1,28_ = 489.946, *p* < 0.001) and *Parp1* deficiency (*F*_1,28_ = 17.651, *p* < 0.001), together with a significant cisplatin × genotype interaction (*F*_1,28_ = 24.576, *p* < 0.001). A similar protective effect of *Parp1* deficiency was observed in male mice at day 4. Cisplatin increased plasma creatinine from 0.23 ± 0.01 to 1.01 ± 0.04 mg/dL in wild-type mice, whereas the increase was attenuated to 0.65 ± 0.02 mg/dL in *Parp1* KO mice (Figure 1C). Two-way ANOVA of logarithmically transformed data revealed significant main effects of cisplatin (*F*_1,28_ = 981.950, *p* < 0.001) and *Parp1* deficiency (*F*_1,28_ = 17.861, *p* < 0.001), as well as a significant cisplatin × genotype interaction (*F*_1,28_ = 48.507, *p* < 0.001). Blood urea nitrogen increased from 21.34 ± 0.73 to 84.65 ± 4.65 mg/dL in wild-type mice and was significantly lower at 53.68 ± 3.36 mg/dL in cisplatin-treated *Parp1* KO mice (Figure 1D). The two-factor linear model with HC3 robust standard errors showed significant main effects of cisplatin (*F*_1,28_ = 531.193, *p* < 0.001) and *Parp1* deficiency (*F*_1,28_ = 20.929, *p* < 0.001), together with a significant cisplatin × genotype interaction (*F*_1,28_ = 20.929, *p* < 0.001).

**Figure 2:**
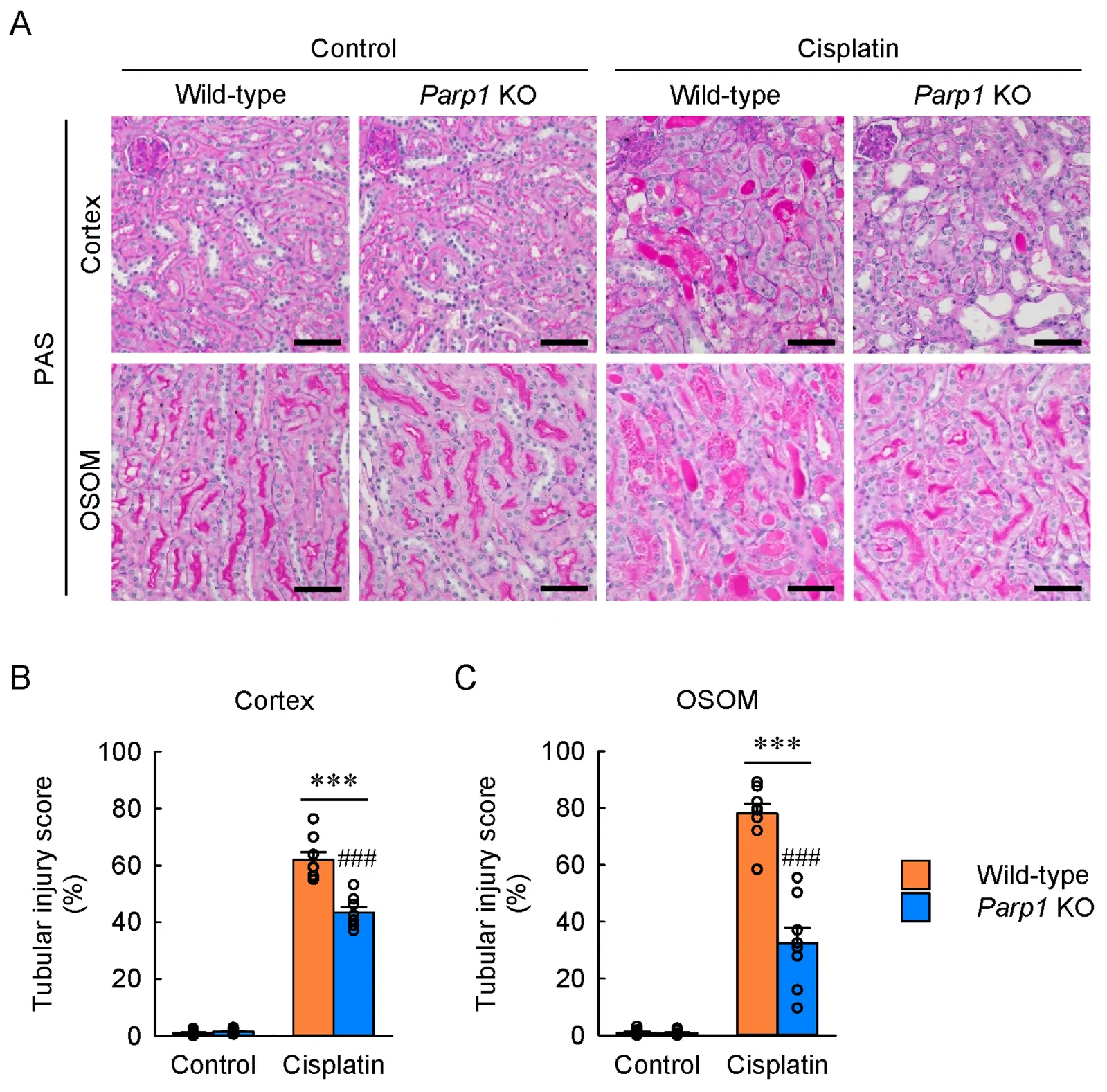
Loss of PARP1 attenuates cisplatin-induced tubular injury. (**A**) Representative images of PAS-stained kidney sections from female wild-type and *Parp1* KO mice 4 days after cisplatin or control treatment. Tubular injury scores were quantified in the (**B**) cortex and (**C**) outer stripe of the outer medulla (OSOM). Scale bar = 50 μm. Data are presented as the mean ± SEM (*n* = 8 per group). Tubular injury scores were square-root-transformed before statistical analysis and analyzed using two-way ANOVA followed by the Holm–Sidak post hoc test. *** *p* < 0.001 versus respective control; ### *p* < 0.001 versus respective wild-type.

Histopathological examination of PAS-stained kidney sections showed marked tubular injury in cisplatin-treated wild-type mice, whereas the injury was less severe in *Parp1* KO mice (Figure 2A). In the renal cortex, cisplatin increased the tubular injury score from 0.94 ± 0.30% to 62.03 ± 2.67% in wild-type mice (Figure 2B). The corresponding injury score was significantly lower in cisplatin-treated *Parp1* KO mice (43.35 ± 1.91%). Two-way ANOVA of square-root-transformed data revealed significant main effects of cisplatin (*F*_1,28_ = 1502.888, *p* < 0.001) and *Parp1* deficiency (*F*_1,28_ = 9.366, *p* = 0.005), as well as a significant cisplatin × genotype interaction (*F*_1,28_ = 24.387, *p* < 0.001). Tubular injury was particularly prominent in the OSOM, where cisplatin increased the injury score from 0.97 ± 0.37% to 78.12 ± 3.43% in wild-type mice (Figure 2C). This increase was significantly attenuated to 32.41 ± 5.50% in *Parp1* KO mice. Two-way ANOVA of square-root-transformed data showed significant main effects of cisplatin (*F*_1,28_ = 417.038, *p* < 0.001) and *Parp1* deficiency (*F*_1,28_ = 29.709, *p* < 0.001), as well as a significant cisplatin × genotype interaction (*F*_1,28_ = 24.909, *p* < 0.001). These findings indicate that loss of PARP1 attenuates cisplatin-induced renal dysfunction and tubular injury.

### 3.2. Loss of PARP1 Attenuates Cisplatin-Induced Renal Inflammatory Gene Expression

**Figure 3:**
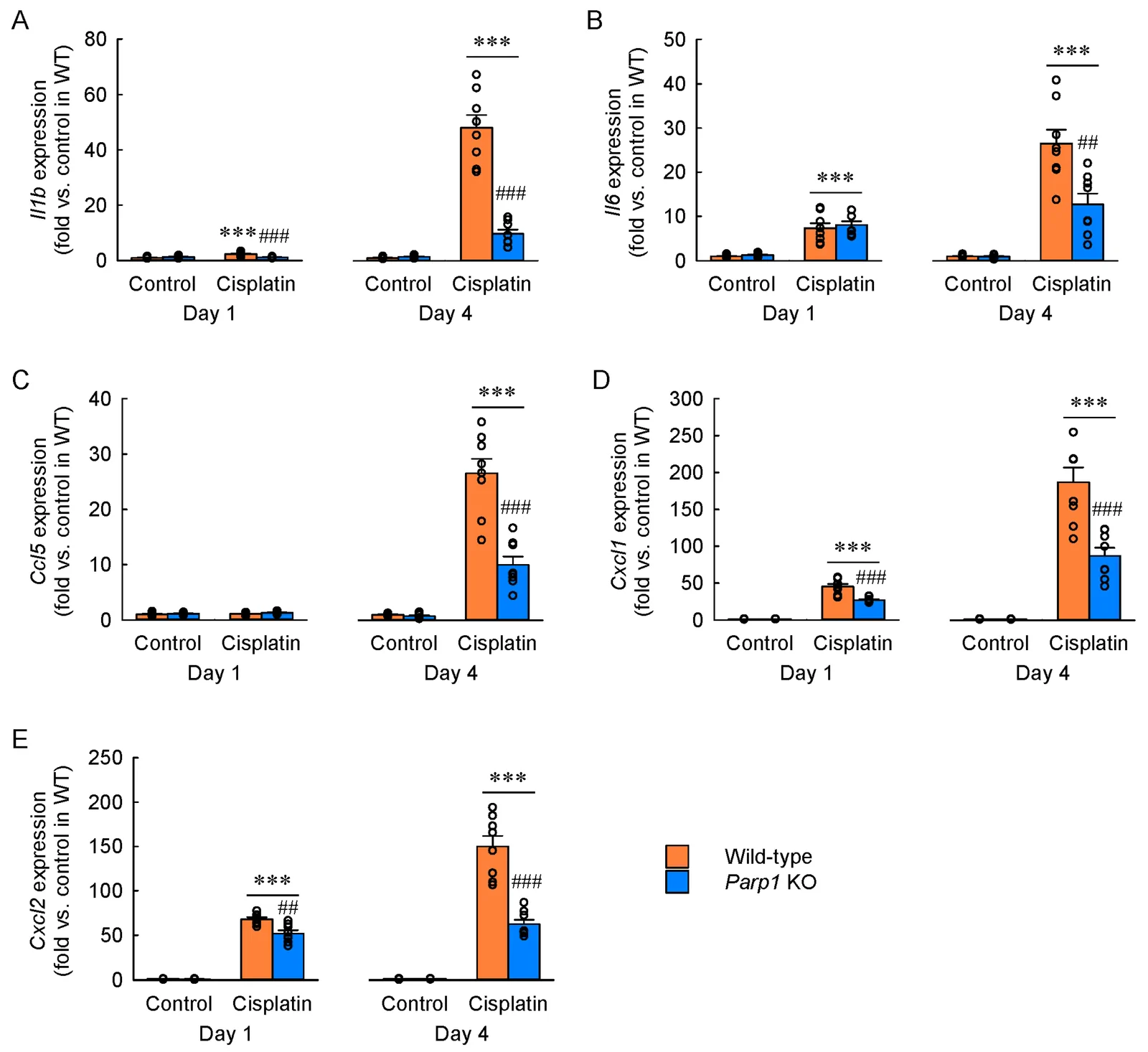
Loss of PARP1 attenuates cisplatin-induced renal inflammatory gene expression. Kidney mRNA expression of (**A**) *Il1b*, (**B**) *Il6*, (**C**) *Ccl5*, (**D**) *Cxcl1*, and (**E**) *Cxcl2* was measured by quantitative real-time PCR in female wild-type and *Parp1* KO mice at days 1 and 4 after cisplatin or control treatment and normalized to the corresponding control wild-type group. Data are presented as the mean ± SEM (*n* = 8 per group). Except for *Ccl5* expression at day 1, all datasets were logarithmically transformed before statistical analysis. *Il6* and *Ccl5* expression at day 4 and *Cxcl2* expression at day 1 were analyzed using a two-factor linear model with HC3 robust standard errors and Type III Wald *F* tests, followed by Holm-adjusted pairwise comparisons. The remaining datasets were analyzed using two-way ANOVA followed by the Holm–Sidak post hoc test. *** *p* < 0.001 versus respective control; ## *p* < 0.01 and ### *p* < 0.001 versus respective wild-type.

We next examined the renal expression of inflammatory cytokine and chemokine genes in wild-type and *Parp1* KO mice at days 1 and 4 after cisplatin administration (Figure 3A–E). At day 1, cisplatin increased *Il1b* expression to 2.29 ± 0.25 fold in wild-type mice, whereas this increase was attenuated in *Parp1* KO mice (1.13 ± 0.08 fold; Figure 3A). Two-way ANOVA of logarithmically transformed data showed significant effects of cisplatin (*F*_1,28_ = 12.572, *p* = 0.001) and *Parp1* deficiency (*F*_1,28_ = 4.534, *p* = 0.042), as well as a significant cisplatin × genotype interaction (*F*_1,28_ = 15.777, *p* < 0.001). At day 4, cisplatin markedly increased *Il1b* mRNA expression to 48.04 ± 4.61 fold relative to control wild-type mice (Figure 3A). This increase was significantly attenuated in *Parp1* KO mice, in which *Il1b* expression reached 9.71 ± 1.49 fold. Two-way ANOVA of logarithmically transformed data showed significant main effects of cisplatin (*F*_1,28_ = 496.933, *p* < 0.001) and *Parp1* deficiency (*F*_1,28_ = 26.561, *p* < 0.001), as well as a significant cisplatin × genotype interaction (*F*_1,28_ = 55.922, *p* < 0.001). *Il6* expression was increased by cisplatin in both wild-type and *Parp1* KO mice at day 1 (7.33 ± 1.16 and 8.06 ± 0.88 fold, respectively; Figure 3B), with a significant main effect of cisplatin (*F*_1,28_ = 219.015, *p* < 0.001), but no significant effect of *Parp1* deficiency (*F*_1,28_ = 1.785, *p* = 0.192) or cisplatin × genotype interaction (*F*_1,28_ = 0.050, *p* = 0.824). At day 4, *Il6* expression increased to 26.46 ± 3.15 fold in cisplatin-treated wild-type mice but was significantly lower in cisplatin-treated *Parp1* KO mice at 12.72 ± 2.39 fold (Figure 3B). A two-factor linear model with HC3 robust standard errors showed significant effects of cisplatin (*F*_1,28_ = 267.196, *p* < 0.001) and *Parp1* deficiency (*F*_1,28_ = 8.207, *p* = 0.004), whereas the cisplatin × genotype interaction was not significant (*F*_1,28_ = 3.377, *p* = 0.066). *Ccl5* expression was not significantly increased by cisplatin at day 1 (Figure 3C). At day 4, cisplatin increased *Ccl5* expression to 26.59 ± 2.60 fold in wild-type mice, whereas expression was significantly lower in cisplatin-treated *Parp1* KO mice (10.01 ± 1.48 fold; Figure 3C). A two-factor linear model with HC3 robust standard errors showed significant effects of cisplatin (*F*_1,28_ = 339.685, *p* < 0.001) and *Parp1* deficiency (*F*_1,28_ = 20.172, *p* < 0.001), while the cisplatin × genotype interaction did not reach statistical significance (*F*_1,28_ = 3.331, *p* = 0.079). At day 1, *Cxcl1* expression increased to 45.07 ± 3.47 fold in cisplatin-treated wild-type mice and was significantly lower in *Parp1* KO mice (26.87 ± 1.28 fold; Figure 3D). Two-way ANOVA of logarithmically transformed data showed significant effects of cisplatin (*F*_1,28_ = 2228.825, *p* < 0.001) and *Parp1* deficiency (*F*_1,28_ = 6.076, *p* = 0.020), together with a significant cisplatin × genotype interaction (*F*_1,28_ = 18.638, *p* < 0.001). At day 4, cisplatin increased *Cxcl1* expression to 187.19 ± 19.99 fold in wild-type mice, whereas this increase was significantly attenuated to 86.89 ± 11.03 fold in *Parp1* KO mice (Figure 3D). Two-way ANOVA revealed significant main effects of cisplatin (*F*_1,28_ = 1978.253, *p* < 0.001) and *Parp1* deficiency (*F*_1,28_ = 12.034, *p* = 0.002), together with a significant cisplatin × genotype interaction (*F*_1,28_ = 14.360, *p* < 0.001). At day 1, *Cxcl2* expression increased to 68.16 ± 2.09 fold in wild-type mice and was significantly lower in *Parp1* KO mice (52.01 ± 3.53 fold; Figure 3E). The two-factor linear model with HC3 robust standard errors showed significant effects of cisplatin (*F*_1,28_ = 915.850, *p* < 0.001) and *Parp1* deficiency (*F*_1,28_ = 12.870, *p* = 0.001), whereas the cisplatin × genotype interaction was not significant (*F*_1,28_ = 0.170, *p* = 0.681). At day 4, cisplatin increased *Cxcl2* expression to 149.70 ± 12.19 fold in wild-type mice, whereas the increase was significantly attenuated to 62.53 ± 4.98 fold in *Parp1* KO mice (Figure 3E). Two-way ANOVA showed significant main effects of cisplatin (*F*_1,28_ = 2631.614, *p* < 0.001) and *Parp1* deficiency (*F*_1,28_ = 13.492, *p* = 0.001), as well as a significant cisplatin × genotype interaction (*F*_1,28_ = 39.300, *p* < 0.001). Collectively, these results indicate that loss of PARP1 attenuates cisplatin-induced renal inflammatory gene expression.

### 3.3. Loss of PARP1 Attenuates Cisplatin-Induced Renal NF-κB Activation and Renal COX-2/PGE2 Response

**Figure 4:**
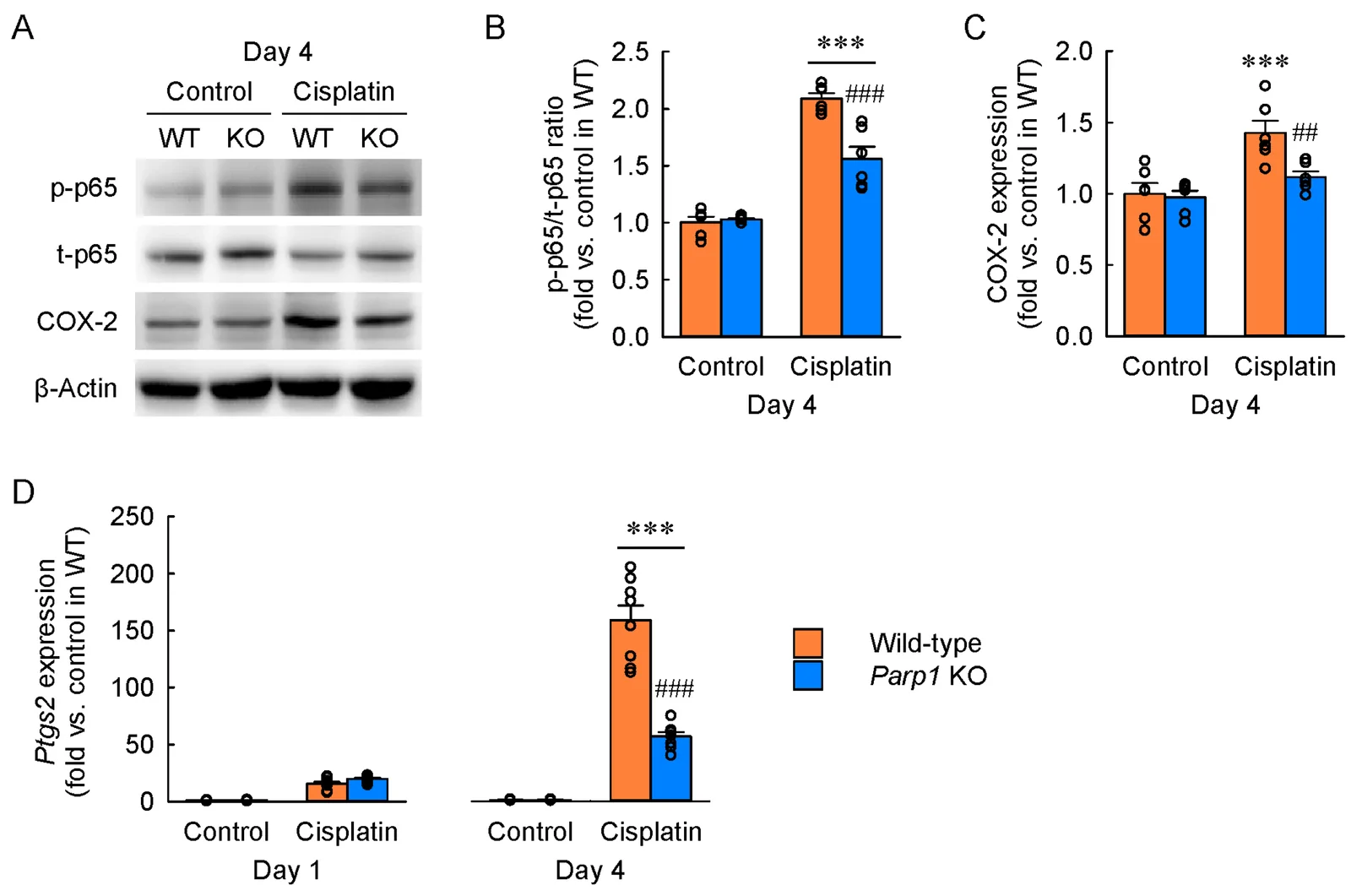
Loss of PARP1 attenuates cisplatin-induced NF-κB activation and increases in renal *Ptgs2* mRNA and COX-2 protein expression. (**A**) Representative Western blot images of phosphorylated NF-κB p65 (p-p65), total NF-κB p65 (t-p65), COX-2, and β-actin in kidney tissues from female wild-type and *Parp1* KO mice 4 days after cisplatin or control treatment. (**B**) Renal NF-κB activation was assessed as the p-p65/t-p65 ratio. (**C**) Densitometric analysis of COX-2 protein expression normalized to β-actin. (**D**) Kidney *Ptgs2* mRNA expression was measured by quantitative real-time PCR in female wild-type and *Parp1* KO mice at days 1 and 4 after cisplatin or control treatment and normalized to the corresponding control wild-type group. Data are presented as the mean ± SEM (*n* = 6 per group for Western blot analyses and *n* = 8 per group for *Ptgs2* expression). Data for *Ptgs2* expression were logarithmically transformed before statistical analysis. The ratio of p-p65 to t-p65 was analyzed using a two-factor linear model with HC3 robust standard errors and Type III Wald *F* tests, followed by Holm-adjusted pairwise comparisons. COX-2 expression and *Ptgs2* expression were analyzed using two-way ANOVA followed by the Holm–Sidak post hoc test. *** *p* < 0.001 versus respective control; ## *p* < 0.01 and ### *p* < 0.001 versus respective wild-type.

**Figure 5:**
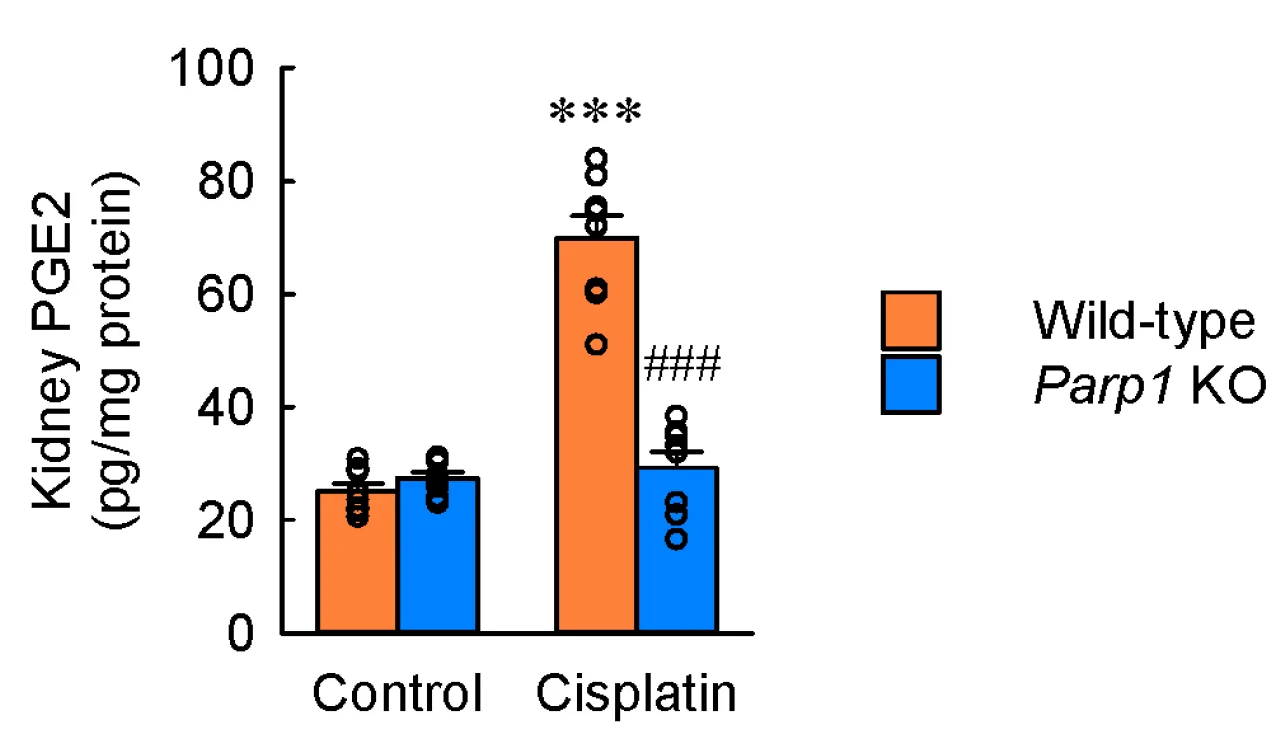
Loss of PARP1 attenuates cisplatin-induced increases in renal PGE2 levels. Prostaglandin E2 (PGE2) levels were measured in kidney tissue homogenates from female wild-type and *Parp1* KO mice 4 days after cisplatin or control treatment and normalized to total protein content. Data are presented as the mean ± SEM (*n* = 8 per group). Data were analyzed using two-way ANOVA followed by the Holm–Sidak post hoc test. *** *p* < 0.001 versus respective control; ### *p* < 0.001 versus respective wild-type.

To further investigate the inflammatory mechanisms associated with PARP1, we examined renal NF-κB activation, COX-2 protein and *Ptgs2* mRNA expression, together with kidney PGE2 levels, as shown in Figure 4 and Figure 5. Western blot analysis showed that cisplatin increased renal NF-κB p65 activation, assessed by the p-p65/t-p65 ratio, at day 4 in wild-type mice (Figure 4A,B). The p-p65/t-p65 ratio increased from 1.00 ± 0.05 to 2.09 ± 0.05 fold in wild-type mice, whereas the increase was significantly attenuated to 1.56 ± 0.11 fold in cisplatin-treated *Parp1* KO mice. A two-factor linear model with HC3 robust standard errors showed significant effects of cisplatin (*F*_1,20_ = 21.045, *p* < 0.001) and *Parp1* deficiency (*F*_1,20_ = 17.253, *p* < 0.001), as well as a significant cisplatin × genotype interaction (*F*_1,20_ = 16.019, *p* < 0.001). Western blot analysis also showed increased renal COX-2 protein expression after cisplatin administration in wild-type mice (Figure 4A,C). COX-2 expression increased from 1.00 ± 0.08 to 1.43 ± 0.09 fold in wild-type mice, whereas no significant cisplatin-induced increase was observed in *Parp1* KO mice (0.98 ± 0.05 fold in control mice versus 1.12 ± 0.04 fold after cisplatin treatment). COX-2 expression after cisplatin treatment was significantly lower in *Parp1* KO mice than in wild-type mice. Two-way ANOVA revealed significant main effects of cisplatin (*F*_1,20_ = 19.063, *p* < 0.001) and *Parp1* deficiency (*F*_1,20_ = 6.562, *p* = 0.019), as well as a significant cisplatin × genotype interaction (*F*_1,20_ = 4.792, *p* = 0.041). We next examined renal *Ptgs2* mRNA expression at days 1 and 4 after cisplatin administration (Figure 4D). At day 1, cisplatin increased renal *Ptgs2* mRNA expression to 15.49 ± 1.82 fold in wild-type mice and 19.93 ± 0.99 fold in *Parp1* KO mice, with no significant difference between the two genotypes after cisplatin treatment (Figure 4D). Two-way ANOVA of logarithmically transformed data showed a significant main effect of cisplatin (*F*_1,28_ = 623.870, *p* < 0.001), but no significant effect of *Parp1* deficiency (*F*_1,28_ = 2.688, *p* = 0.112) or cisplatin × genotype interaction (*F*_1,28_ = 1.002, *p* = 0.325). At day 4, cisplatin markedly increased renal *Ptgs2* mRNA expression to 158.62 ± 12.92 fold relative to control wild-type mice (Figure 4D). In cisplatin-treated *Parp1* KO mice, *Ptgs2* expression was significantly lower at 56.71 ± 3.76 fold. Two-way ANOVA of logarithmically transformed data revealed significant main effects of cisplatin (*F*_1,28_ = 1575.089, *p* < 0.001) and *Parp1* deficiency (*F*_1,28_ = 18.384, *p* = 0.001), as well as a significant cisplatin × genotype interaction (*F*_1,28_ = 20.591, *p* < 0.001).

We next determined whether these changes in COX-2/*Ptgs2* expression were accompanied by alterations in renal PGE2 levels (Figure 5). Cisplatin increased kidney PGE2 levels from 25.11 ± 1.37 to 69.87 ± 4.03 pg/mg protein in wild-type mice. In contrast, PGE2 levels remained near baseline in cisplatin-treated *Parp1* KO mice at 29.27 ± 2.82 pg/mg protein and were significantly lower than those in cisplatin-treated wild-type mice. Two-way ANOVA showed significant main effects of cisplatin (*F*_1,28_ = 79.924, *p* < 0.001) and *Parp1* deficiency (*F*_1,28_ = 53.945, *p* < 0.001), as well as a significant cisplatin × genotype interaction (*F*_1,28_ = 67.564, *p* < 0.001). Together, these findings demonstrate that PARP1 deficiency is associated with marked attenuation of the renal COX-2/PGE2 response to cisplatin.

## 4. Discussion

In the present study, loss of PARP1 markedly attenuated cisplatin-induced acute kidney injury, as evidenced by improved renal function, reduced tubular injury, suppressed inflammatory gene expression, and attenuation of the renal COX-2/PGE2 response. These findings are consistent with and extend our previous observations that PARP1 contributes to cisplatin nephrotoxicity [25,32,45]. Importantly, whereas the renoprotective and anti-inflammatory effects of PARP1 deficiency have been established previously, the present study newly identifies the renal COX-2/PGE2 response as a molecular component associated with PARP1-dependent cisplatin nephrotoxicity. Consistent with this protective phenotype, *Parp1* KO mice exhibited significantly lower plasma creatinine and BUN levels than wild-type mice following cisplatin administration, and tubular injury was reduced in both the renal cortex and the OSOM. Elevations in circulating creatinine and BUN are commonly used indices of renal dysfunction in single-dose cisplatin AKI models, and the magnitude and timing observed here are consistent with functional impairment reported in comparable experimental settings [56,57,58]. The greater extent of tubular injury observed in the OSOM than in the cortex is consistent with the well-established vulnerability of the S3 segment of the proximal tubule, which is concentrated in the outer medulla and is particularly susceptible to cisplatin accumulation and toxicity [59,60,61]. A recent spatiotemporal transcriptomic study similarly demonstrated that outer-medullary proximal tubules exhibit stronger and more sustained DNA damage and inflammatory transcriptional responses to cisplatin than cortical proximal tubules, further supporting regional heterogeneity in cisplatin nephrotoxicity [62]. Our finding that PARP1 deficiency attenuated tubular injury in both the cortex and OSOM suggests that PARP1-dependent mechanisms contribute to cisplatin-induced tubular damage across anatomically distinct proximal tubular regions.

Cisplatin administration markedly increased renal expression of pro-inflammatory cytokines and chemokines, and this induction was significantly attenuated in *Parp1* KO mice. Among the cytokines examined, *Il1b* and *Il6* were markedly upregulated by cisplatin, consistent with their established roles as early inflammatory mediators that amplify tubular inflammation following cisplatin-induced cell injury [63,64,65]. The chemokines *Ccl5*, *Cxcl1*, and *Cxcl2*, in contrast, contribute to the recruitment of circulating immune cells into the injured kidney. *Cxcl1* and *Cxcl2* are potent neutrophil chemoattractants and are strongly induced during cisplatin nephrotoxicity, whereas *Ccl5* has been implicated in NF-κB-dependent tubular inflammatory signaling and macrophage polarization [11,66]. The coordinated attenuation of these inflammatory genes in *Parp1* KO mice is consistent with a role for PARP1 in NF-κB-dependent inflammatory transcription [67,68,69]. Importantly, at day 1 after cisplatin administration, when plasma creatinine and blood urea nitrogen levels did not differ significantly between genotypes, the induction of *Il1b*, *Cxcl1*, and *Cxcl2* was already significantly attenuated in *Parp1* KO mice. This temporal dissociation suggests that suppression of at least a subset of inflammatory responses is not solely secondary to the lower degree of renal dysfunction observed at day 4. However, because *Il6* and *Ptgs2* did not show significant genotype-dependent differences at day 1, these findings do not establish a generalized or direct anti-inflammatory effect of PARP1 deficiency. We previously showed that renal NF-κB activation, neutrophil infiltration, and pro-inflammatory gene expression were reduced in *Parp1*-deficient kidneys following cisplatin injury [25]. Pharmacological PARP inhibition has likewise been reported to attenuate NF-κB activation and NF-κB-dependent inflammatory gene expression [70,71,72]. Together, these findings support a model in which PARP1 facilitates cisplatin-induced NF-κB signaling and contributes to the associated cytokine and chemokine response during kidney injury.

An important new finding of the present study is that PARP1 deficiency is associated with marked attenuation of the renal COX-2/PGE2 response to cisplatin. Cisplatin administration markedly increased renal *Ptgs2* mRNA expression, COX-2 protein abundance, and kidney PGE2 levels in wild-type mice, consistent with previous evidence implicating COX-2-dependent prostaglandin production in experimental cisplatin nephrotoxicity [38,73,74]. Importantly, previous studies have provided functional evidence for the involvement of this pathway in cisplatin nephrotoxicity. Genetic deletion of microsomal prostaglandin E synthase-1 (mPGES-1) or pharmacological inhibition of COX-2 with celecoxib attenuated cisplatin-induced renal dysfunction, structural injury, and inflammatory responses in mice, and celecoxib similarly reduced renal functional and histological injury in a rat model [37,75]. In the present study, cisplatin-induced increases in *Ptgs2* mRNA expression and COX-2 protein abundance were substantially attenuated in *Parp1* KO mice, while the increase in kidney PGE2 levels was nearly abolished. The concordant attenuation of *Ptgs2* mRNA, COX-2 protein, and PGE2 levels demonstrates that loss of PARP1 is associated with suppression of multiple components of the renal COX-2/PGE2 response. A mechanistic link between PARP1 and COX-2 is biologically plausible because *Ptgs2* transcription is regulated in part by NF-κB-dependent signaling, while PARP1 facilitates NF-κB-dependent transcription [76,77,78]. Together with the present finding that cisplatin-induced renal NF-κB activation was attenuated in *Parp1*-deficient kidneys, these results raise the possibility of a PARP1–NF-κB–COX-2/PGE2 signaling axis contributing to cisplatin-induced renal inflammation. However, the current study does not establish a direct causal relationship among these components. We did not determine whether PARP1 directly regulates the *Ptgs2* promoter or whether selective inhibition or restoration of COX-2/PGE2 signaling alters the protective phenotype of *Parp1* deficiency. Accordingly, the reduction in COX-2 and PGE2 observed in *Parp1* KO mice should be interpreted as an association rather than as conclusive evidence that suppression of this pathway mediates the attenuation of renal inflammation and injury. Similarly, the cisplatin-induced increase in renal PGE2 should be regarded as a biochemical response associated with COX-2 activation rather than as direct evidence that PGE2 itself promotes nephrotoxicity.

This study has several limitations. First, the relationship between PARP1 and the COX-2/PGE2 pathway was evaluated using genetic loss of PARP1 without direct pharmacological manipulation of the COX-2/PGE2 pathway. Second, although renal NF-κB activation was assessed by p65 phosphorylation, the present study did not directly establish a causal link between reduced NF-κB activation and suppression of the COX-2/PGE2 response in *Parp1*-deficient kidneys. The proposed PARP1–NF-κB–COX-2/PGE2 axis therefore remains associative rather than causally established. Third, *Ptgs2* mRNA expression, COX-2 protein abundance, inflammatory gene expression, and PGE2 levels were measured in whole-kidney tissue. Therefore, the present study cannot identify the specific renal cell populations responsible for the altered COX-2/PGE2 response in *Parp1* KO mice. The observed changes may reflect responses in tubular epithelial cells, infiltrating immune cells, interstitial cells, or other renal compartments. Cell-type-specific localization of COX-2 and related signaling will be required to define the cellular source of this response. Fourth, PGE2 signals through four receptor subtypes (EP1–EP4) with context-dependent and sometimes opposing effects on renal inflammation [79,80,81]. Accordingly, the present findings do not establish whether broad COX-2 inhibition or selective targeting of a specific EP receptor would represent the more appropriate therapeutic strategy. Finally, although attenuation of cisplatin-induced renal dysfunction was observed in both male and female mice, all histological and mechanistic analyses were performed in female mice. Therefore, potential sex-dependent differences in the underlying molecular mechanisms were not comprehensively evaluated. The study was also restricted to the acute phase after a single cisplatin dose, with temporal analyses limited to selected endpoints at days 1 and 4. Because *Ptgs2* expression was not significantly different between genotypes at day 1, the present study does not establish that suppression of the COX-2/PGE2 response precedes the development of renal protection. Whether these mechanistic findings extend to male mice, repeated cisplatin regimens, or the progression from acute to chronic cisplatin-induced kidney injury remains to be determined. Although the cisplatin-induced AKI model reproduces key features of cisplatin nephrotoxicity, the extent to which the present findings translate to human cisplatin-associated AKI remains to be established.

## 5. Conclusions

This study identifies attenuation of the renal COX-2/PGE2 response as a novel feature of the protective phenotype associated with PARP1 deficiency during cisplatin-induced AKI. Loss of PARP1 reduced renal dysfunction, tubular injury, inflammatory gene expression, *Ptgs2* transcription, COX-2 protein expression, and renal PGE2 levels. Together, these findings support a potential PARP1–NF-κB–COX-2/PGE2 signaling axis in cisplatin nephrotoxicity and warrant further studies to determine its causal and therapeutic significance.

## Data Availability

The original data presented in this study are included in the article and Supplementary File S1. Further inquiries can be directed to the corresponding author.

## Supplementary Materials

The following supporting information can be downloaded at: https://www.mdpi.com/article/10.3390/cells15191810/s1. Supplementary File S1: Supplementary_Raw_Data.pdf.

## Abbreviations

The following abbreviations are used in this manuscript:

AKI	Acute kidney injury
ARRIVE	Animal Research: Reporting of In Vivo Experiments
ATP	Adenosine triphosphate
BUN	Blood urea nitrogen
COX-2	Cyclooxygenase-2
ELISA	Enzyme-linked immunosorbent assay
HC3	Heteroskedasticity-consistent covariance matrix estimator type 3
IACUC	Institutional Animal Care and Use Committee
KO	Knockout
NAD^+^	Nicotinamide adenine dinucleotide
NF-κB	Nuclear factor kappa B
OSOM	Outer stripe of the outer medulla
PARP1	Poly(ADP-ribose) polymerase 1
PAS	Periodic acid–Schiff
PGE2	Prostaglandin E2
qRT-PCR	Quantitative real-time polymerase chain reaction
SEM	Standard error of the mean
TLR4	Toll-like receptor 4
WT	Wild-type
