# Supplementary material for: PARP1 Deficiency Attenuates Cisplatin Nephrotoxicity and Renal COX-2/PGE2 Response
Source: Cells. 2026 Oct 3;15(19):1810. doi: 10.3390/cells15191810 (PMC13664194; doi:10.3390/cells15191810)
Supplement: Supplementary file 1 [file cells-15-01810-s001.zip › cells-4548157-supplementary.pdf]

PLASMA CREATININE IN FEMALE (DAY 1)

mg/dL

Kidney	Control		Cisplatin	
no.	WT	*Parp1* KO	WT	*Parp1* KO
1	0.26	0.21	0.32	0.35
2	0.28	0.18	0.31	0.37
3	0.25	0.25	0.30	0.34
4	0.23	0.28	0.36	0.31
5	0.30	0.27	0.34	0.32
6	0.25	0.23	0.27	0.39
7	0.28	0.29	0.30	0.38
8	0.26	0.25	0.37	0.33
**mean**	**0.26**	**0.24**	**0.32**	**0.35**
**SD**	**0.02**	**0.04**	**0.03**	**0.03**
**SEM**	**0.01**	**0.01**	**0.01**	**0.01**
vs. WT		0.232		0.065
vs. control			<0.001	<0.001

Normality Test (Shapiro-Wilk) : Passed (p = 0.574)

Equal Variance Test (Brown-Forsythe) : Passed (p = 0.623)

Two Way ANOVA

Source of Variation	DF	SS	MS	*F*	*p*
Cisplatin	1	0.0525	0.0525	56.796	<0.001
Parp1 KO	1	0.000228	0.000228	0.246	0.624
Cisplatin x KO	1	0.00457	0.00457	4.941	0.034
Residual	28	0.0259	0.000925		
Total	31	0.0832	0.00268		

PLASMA CREATININE IN FEMALE (DAY 4)

mg/dL

Kidney	Control		Cisplatin	
no.	WT	*Parp1* KO	WT	*Parp1* KO
1	0.20	0.27	1.13	0.73
2	0.17	0.27	0.80	0.48
3	0.26	0.26	1.15	0.58
4	0.29	0.24	0.81	0.62
5	0.28	0.29	0.89	0.67
6	0.24	0.24	0.94	0.56
7	0.28	0.27	0.96	0.59
8	0.26	0.27	1.04	0.63
**mean**	**0.25**	**0.27**	**0.96**	**0.61**
**SD**	**0.04**	**0.02**	**0.13**	**0.07**
**SEM**	**0.01**	**0.01**	**0.05**	**0.03**

Logarithmic transformation

Kidney	Control		Cisplatin	
no.	WT	*Parp1* KO	WT	*Parp1* KO
1	-0.70	-0.56	0.05	-0.14
2	-0.76	-0.58	-0.10	-0.32
3	-0.58	-0.58	0.06	-0.24
4	-0.54	-0.62	-0.09	-0.21
5	-0.55	-0.54	-0.05	-0.18
6	-0.62	-0.62	-0.03	-0.25
7	-0.56	-0.56	-0.02	-0.23
8	-0.58	-0.56	0.02	-0.20
**mean**	**-0.61**	**-0.58**	**-0.02**	**-0.22**
**SD**	**0.08**	**0.03**	**0.06**	**0.05**
**SEM**	**0.03**	**0.01**	**0.02**	**0.02**
vs. WT		0.228		<0.001
vs. control			<0.001	<0.001

Normality Test (Shapiro-Wilk) : Passed (p = 0.278)

Equal Variance Test (Brown-Forsythe) : Passed (p = 0.345)

Two Way ANOVA

Source of Variation	DF	SS	MS	*F*	*p*
Cisplatin	1	1.808	1.808	538.915	<0.001
Parp1 KO	1	0.0540	0.0540	16.089	<0.001
Cisplatin x KO	1	0.111	0.111	33.109	<0.001
Residual	28	0.0939	0.00335		
Total	31	2.067	0.0667		

BLOOD UREA NITROGEN IN FEMALE (DAY 1)

mg/dL

Kidney	Control		Cisplatin	
no.	WT	*Parp1* KO	WT	*Parp1* KO
1	20.9	20.1	26.6	25.0
2	20.6	21.9	24.3	23.8
3	20.1	19.0	26.9	25.3
4	20.8	22.8	25.7	22.8
5	21.2	17.7	20.3	23.5
6	19.7	16.7	23.3	24.1
7	20.4	19.5	24.8	25.0
8	21.4	23.1	26.3	26.7
**mean**	**20.6**	**20.1**	**24.8**	**24.5**
**SD**	**0.6**	**2.3**	**2.2**	**1.2**
**SEM**	**0.2**	**0.8**	**0.8**	**0.4**
vs. WT		1.000		1.000
vs. control			<0.001	<0.001

Normality Test (Shapiro-Wilk) : Passed (p = 0.478)

Equal Variance Test (Brown-Forsythe) : Failed (p < 0.050)

Two-factor linear model with HC3 robust SE (Type III Wald *F*)

Source of Variation	DF	Wald *F*	*p*
Cisplatin	1, 28	42.460	<0.001
Parp1 KO	1, 28	0.359	0.554
Cisplatin x KO	1, 28	0.048	0.828

BLOOD UREA NITROGEN IN FEMALE (DAY 4)

mg/dL

Kidney	Control		Cisplatin	
no.	WT	*Parp1* KO	WT	*Parp1* KO
1	20.7	21.3	77.3	34.9
2	21.9	20.9	104.6	65.5
3	18.9	20.2	77.5	43.4
4	22.8	21.2	77.2	48.8
5	17.7	21.2	91.6	59.6
6	16.7	19.7	88.2	58.4
7	19.4	20.7	61.2	48.8
8	23.5	21.5	70.0	48.5
**mean**	**20.2**	**20.9**	**80.9**	**51.0**
**SD**	**2.4**	**0.6**	**13.5**	**9.8**
**SEM**	**0.9**	**0.2**	**4.8**	**3.5**

Logarithmic transformation

Kidney	Control		Cisplatin	
no.	WT	*Parp1* KO	WT	*Parp1* KO
1	1.32	1.33	1.89	1.54
2	1.34	1.32	2.02	1.82
3	1.28	1.31	1.89	1.64
4	1.36	1.33	1.89	1.69
5	1.25	1.33	1.96	1.78
6	1.22	1.29	1.95	1.77
7	1.29	1.32	1.79	1.69
8	1.37	1.33	1.85	1.69
**mean**	**1.30**	**1.32**	**1.90**	**1.70**
**SD**	**0.05**	**0.01**	**0.07**	**0.09**
**SEM**	**0.02**	**0.00**	**0.03**	**0.03**
vs. WT		0.597		<0.001
vs. control			<0.001	<0.001

Normality Test (Shapiro-Wilk) : Passed (p = 0.298)

Equal Variance Test (Brown-Forsythe) : Passed (p = 0.083)

Two Way ANOVA

Source of Variation	DF	SS	MS	*F*	*p*
Cisplatin	1	1.926	1.926	489.946	<0.001
Parp1 KO	1	0.0694	0.0694	17.651	<0.001
Cisplatin x KO	1	0.0966	0.0966	24.576	<0.001
Residual	28	0.110	0.00393		
Total	31	2.202	0.0710		

PLASMA CREATININE IN MALE (DAY 4)

mg/dL

Kidney no.	Control WT	Control *Parp1* KO	Cisplatin WT	Cisplatin *Parp1* KO
1	0.18	0.27	1.17	0.73
2	0.21	0.22	0.86	0.56
3	0.25	0.26	1.08	0.62
4	0.27	0.24	0.91	0.67
5	0.22	0.29	1.00	0.70
6	0.19	0.21	0.94	0.58
7	0.26	0.25	1.05	0.64
8	0.24	0.26	1.09	0.68
**mean**	**0.23**	**0.25**	**1.01**	**0.65**
**SD**	**0.03**	**0.02**	**0.10**	**0.06**
**SEM**	**0.01**	**0.01**	**0.04**	**0.02**

Logarithmic transformation

Kidney no.	Control WT	Control *Parp1* KO	Cisplatin WT	Cisplatin *Parp1* KO
1	-0.75	-0.57	0.07	-0.14
2	-0.68	-0.65	-0.06	-0.25
3	-0.61	-0.59	0.03	-0.21
4	-0.57	-0.62	-0.04	-0.17
5	-0.66	-0.54	0.00	-0.15
6	-0.73	-0.67	-0.03	-0.23
7	-0.59	-0.60	0.02	-0.19
8	-0.62	-0.59	0.04	-0.17
**mean**	**-0.65**	**-0.60**	**0.00**	**-0.19**
**SD**	**0.06**	**0.04**	**0.04**	**0.04**
**SEM**	**0.02**	**0.01**	**0.02**	**0.01**
vs. WT		0.063		<0.001
vs. control			<0.001	<0.001

Normality Test (Shapiro-Wilk) : Passed (p = 0.471)

Equal Variance Test (Brown-Forsythe) : Passed (p = 0.294)

Two Way ANOVA

Source of Variation	DF	SS	MS	*F*	*p*
Cisplatin	1	2.296	2.296	981.950	<0.001
Parp1 KO	1	0.0418	0.0418	17.861	<0.001
Cisplatin x KO	1	0.113	0.113	48.507	<0.001
Residual	28	0.0655	0.00234		
Total	31	2.517	0.0812		

BLOOD UREA NITROGEN IN MALE (DAY 4)

mg/dL

Kidney no.	Control WT	Control *Parp1* KO	Cisplatin WT	Cisplatin *Parp1* KO
1	21.6	21.5	81.7	38.2
2	24.4	20.3	108.1	67.5
3	19.5	21.9	80.6	45.6
4	23.6	21.1	74.1	52.2
5	18.4	20.6	95.3	62.9
6	20.0	22.2	89.2	59.6
7	22.4	20.8	65.0	53.6
8	20.8	21.4	83.2	49.9
**mean**	**21.3**	**21.2**	**84.6**	**53.7**
**SD**	**2.1**	**0.6**	**13.1**	**9.5**
**SEM**	**0.7**	**0.2**	**4.6**	**3.4**

Logarithmic transformation

Kidney no.	Control WT	Control *Parp1* KO	Cisplatin WT	Cisplatin *Parp1* KO
1	1.33	1.33	1.91	1.58
2	1.39	1.31	2.03	1.83
3	1.29	1.34	1.91	1.66
4	1.37	1.32	1.87	1.72
5	1.26	1.31	1.98	1.80
6	1.30	1.35	1.95	1.78
7	1.35	1.32	1.81	1.73
8	1.32	1.33	1.92	1.70
**mean**	**1.33**	**1.33**	**1.92**	**1.72**
**SD**	**0.04**	**0.01**	**0.07**	**0.08**
**SEM**	**0.01**	**0.00**	**0.02**	**0.03**
vs. WT		1.000		<0.001
vs. control			<0.001	<0.001

Normality Test (Shapiro-Wilk) : Passed (p = 0.347)

Equal Variance Test (Brown-Forsythe) : Failed (p < 0.050)

Two-factor linear model with HC3 robust SE (Type III Wald *F*)

Source of Variation	DF	Wald *F*	*p*
Cisplatin	1, 28	531.193	<0.001
Parp1 KO	1, 28	20.929	<0.001
Cisplatin x KO	1, 28	20.929	<0.001

TUBULAR INJURY SCORE IN CORTEX (DAY 4)

Tubular injury score per image

Image no.	Kidney no.	Control						Cisplatin						
		Wild-type			*Parp1* KO			Wild-type			*Parp1* KO			
		injured	total	%	injured	total	%	injured	total	%	injured	total	%	
1		0	32	0.0	0	27	0.0	28	33	84.8	17	25	68.0	
2		0	23	0.0	1	23	4.3	18	34	52.9	12	26	46.2	
3	1	0	25	0.0	1	26	3.8	20	33	60.6	13	33	39.4	
4		0	29	0.0	1	28	3.6	20	35	57.1	8	25	32.0	
5		0	26	0.0	0	30	0.0	21	33	63.6	9	34	26.5	
6		0	23	0.0	1	31	3.2	16	39	41.0	11	36	30.6	
7		0	33	0.0	1	28	3.6	31	32	96.9	12	30	40.0	
8	2	0	38	0.0	1	28	3.6	15	38	39.5	17	35	48.6	
9		1	30	3.3	0	30	0.0	15	37	40.5	17	42	40.5	
10		0	26	0.0	1	26	3.8	20	35	57.1	8	32	25.0	
11		0	23	0.0	0	26	0.0	14	29	48.3	7	27	25.9	
12		0	25	0.0	0	31	0.0	17	29	58.6	15	29	51.7	
13	3	1	34	2.9	1	28	3.6	13	28	46.4	14	25	56.0	
14		0	34	0.0	1	31	3.2	24	37	64.9	15	41	36.6	
15		1	24	4.2	0	30	0.0	25	40	62.5	10	42	23.8	
16		0	27	0.0	0	38	0.0	26	40	65.0	20	35	57.1	
17		0	28	0.0	0	31	0.0	27	29	93.1	11	31	35.5	
18	4	1	30	3.3	1	27	3.7	31	37	83.8	8	25	32.0	
19		0	35	0.0	0	25	0.0	22	25	88.0	21	30	70.0	
20		0	27	0.0	0	29	0.0	15	29	51.7	10	28	35.7	
21		0	28	0.0	1	31	3.2	22	39	56.4	7	30	23.3	
22		1	24	4.2	1	27	3.7	20	33	60.6	23	39	59.0	
23	5	1	25	4.0	0	34	0.0	19	29	65.5	6	40	15.0	
24		1	24	4.2	0	33	0.0	24	29	82.8	15	29	51.7	
25		0	28	0.0	0	32	0.0	27	32	84.4	20	37	54.1	
21		0	23	0.0	1	38	2.6	14	35	40.0	9	33	27.3	
22		0	27	0.0	0	28	0.0	15	30	50.0	10	35	28.6	
23	6	0	30	0.0	0	26	0.0	16	33	48.5	19	25	76.0	
24		0	24	0.0	0	28	0.0	17	29	58.6	13	26	50.0	
25		1	29	3.4	0	35	0.0	29	29	100.0	8	37	21.6	
21		0	30	0.0	0	27	0.0	14	33	42.4	21	26	80.8	
22		0	26	0.0	1	35	2.9	19	28	67.9	24	30	80.0	
23	7	0	25	0.0	0	32	0.0	16	39	41.0	13	39	33.3	
24		0	26	0.0	0	45	0.0	22	38	57.9	7	37	18.9	
25		0	24	0.0	1	34	2.9	24	33	72.7	8	29	27.6	
26		1	26	3.8	1	42	2.4	15	30	50.0	13	28	46.4	16.00
27		0	30	0.0	0	29	0.0	18	38	47.4	18	29	62.1	21.00
28	8	0	39	0.0	0	33	0.0	24	35	68.6	17	29	58.6	20.00
29		0	25	0.0	0	29	0.0	17	34	50.0	9	30	30.0	12.00
30		1	24	4.2	0	27	0.0	28	35	80.0	24	35	68.6	27.00
**mean**		**0.3**	**27.7**	**0.9**	**0.4**	**30.5**	**1.4**	**20.5**	**33.3**	**62.0**	**13.5**	**31.9**	**43.3**	

Tubular injury score per kindey

Kidney no.	Control		Cisplatin	
	Wild-type	*Parp1* KO	Wild-type	*Parp1* KO
1	0.0	2.4	63.8	42.4
2	0.7	2.8	55.0	36.9
3	1.4	1.4	56.1	38.8
4	0.7	0.7	76.3	46.1
5	2.5	1.4	69.9	40.6
6	0.7	0.5	59.4	40.7
7	0.0	1.2	56.4	48.1
8	1.6	0.5	59.2	53.1
**mean**	**0.9**	**1.4**	**62.0**	**43.3**
**SD**	**0.8**	**0.9**	**7.6**	**5.4**
**SEM**	**0.3**	**0.3**	**2.7**	**1.9**

Square-root transformation

Kidney no.	Control		Cisplatin	
	Wild-type	*Parp1* KO	Wild-type	*Parp1* KO
1	0.0	1.5	8.0	6.5
2	0.8	1.7	7.4	6.1
3	1.2	1.2	7.5	6.2
4	0.8	0.9	8.7	6.8
5	1.6	1.2	8.4	6.4
6	0.8	0.7	7.7	6.4
7	0.0	1.1	7.5	6.9
8	1.3	0.7	7.7	7.3
**mean**	**0.8**	**1.1**	**7.9**	**6.6**
**SD**	**0.6**	**0.4**	**0.5**	**0.4**
**SEM**	**0.2**	**0.1**	**0.2**	**0.1**
vs. WT		0.195		<0.001
vs. control			<0.001	<0.001

Normality Test (Shapiro-Wilk) : Passed (p = 0.613)

Equal Variance Test (Brown-Forsythe) : Passed (p = 0.860)

Two Way ANOVA

Source of Variation	DF	SS	MS	*F*	*p*
Cisplatin	1	313.027	313.027	1502.888	<0.001
Parp1 KO	1	1.951	1.951	9.366	0.005
Cisplatin x KO	1	5.079	5.079	24.387	<0.001
Residual	28	5.832	0.208		
Total	31	325.889	10.513		

TUBULAR INJURY SCORE IN OSOM (DAY 4)

Tubular injury score per image

Image no.	Kidney no.	Control						Cisplatin					
		Wild-type			*Parp1* KO			Wild-type			*Parp1* KO		
		injured	total	%	injured	total	%	injured	total	%	injured	total	%
1		0	37	0.0	0	26	0.0	23	30	76.7	15	35	42.9
2		1	43	2.3	1	36	2.8	21	33	63.6	18	29	62.1
3	1	0	36	0.0	1	24	4.2	21	32	65.6	16	29	55.2
4		0	29	0.0	0	35	0.0	29	29	100.0	19	38	50.0
5		0	39	0.0	2	38	5.3	20	37	54.1	12	29	41.4
6		1	35	2.9	0	29	0.0	28	37	75.7	12	33	36.4
7		0	37	0.0	0	37	0.0	30	37	81.1	7	33	21.2
8	2	0	30	0.0	0	47	0.0	21	26	80.8	11	30	36.7
9		0	29	0.0	1	35	2.9	30	37	81.1	14	35	40.0
10		0	40	0.0	0	35	0.0	20	31	64.5	11	39	28.2
11		1	28	3.6	0	34	0.0	35	37	94.6	10	34	29.4
12		2	38	5.3	0	37	0.0	28	35	80.0	12	33	36.4
13	3	1	34	2.9	0	40	0.0	24	30	80.0	9	33	27.3
14		1	30	3.3	0	33	0.0	27	34	79.4	10	34	29.4
15		0	35	0.0	0	31	0.0	24	37	64.9	11	35	31.4
16		1	29	3.4	0	30	0.0	26	28	92.9	9	28	32.1
17		0	31	0.0	1	30	3.3	22	35	62.9	12	35	34.3
18	4	1	31	3.2	0	30	0.0	29	31	93.5	8	38	21.1
19		0	29	0.0	0	32	0.0	28	29	96.6	10	29	34.5
20		0	38	0.0	0	42	0.0	30	30	100.0	5	29	17.2
21		0	30	0.0	0	28	0.0	31	34	91.2	5	33	15.2
22		0	40	0.0	0	31	0.0	27	38	71.1	9	29	31.0
23	5	0	39	0.0	0	38	0.0	35	35	100.0	3	32	9.4
24		0	27	0.0	0	34	0.0	28	37	75.7	9	34	26.5
25		1	35	2.9	0	36	0.0	25	25	100.0	-1	38	-2.6
21		0	30	0.0	0	34	0.0	22	38	57.9	6	33	18.2
22		0	32	0.0	0	27	0.0	27	33	81.8	2	35	5.7
23	6	0	34	0.0	1	30	3.3	24	31	77.4	2	31	6.5
24		0	27	0.0	0	39	0.0	29	31	93.5	3	28	10.7
25		0	27	0.0	0	31	0.0	27	27	100.0	2	30	6.7
21		0	38	0.0	0	30	0.0	23	28	82.1	14	32	43.8
22		0	40	0.0	0	42	0.0	25	30	83.3	7	35	20.0
23	7	0	28	0.0	0	36	0.0	21	37	56.8	18	33	54.5
24		0	37	0.0	0	33	0.0	20	27	74.1	10	35	28.6
25		0	24	0.0	0	43	0.0	28	28	100.0	16	42	38.1
26		0	27	0.0	0	49	0.0	20	30	66.7	20	35	57.1
27		1	29	3.4	0	25	0.0	19	38	50.0	15	38	39.5
28	8	1	37	2.7	1	34	2.9	17	29	58.6	20	33	60.6
29		1	36	2.8	1	33	3.0	23	42	54.8	18	33	54.5
30		0	31	0.0	1	38	2.6	18	29	62.1	19	29	65.5
**mean**		**0.3**	**33.2**	**1.0**	**0.3**	**34.3**	**0.8**	**25.1**	**32.6**	**78.1**	**10.7**	**33.2**	**32.4**

Tubular injury score per kindey

Kidney no.	Control		Cisplatin	
	Wild-type	*Parp1* KO	Wild-type	*Parp1* KO
1	0.5	2.4	72.0	50.3
2	0.6	0.6	76.6	32.5
3	3.0	0.0	79.8	30.8
4	1.3	0.7	89.2	27.8
5	0.6	0.0	87.6	15.9
6	0.0	0.7	82.1	9.5
7	0.0	0.0	79.3	37.0
8	1.8	1.7	58.4	55.5
**mean**	**1.0**	**0.8**	**78.1**	**32.4**
**SD**	**1.0**	**0.9**	**9.7**	**15.5**
**SEM**	**0.4**	**0.3**	**3.4**	**5.5**

Square-root transformation

Kidney no.	Control		Cisplatin	
	Wild-type	*Parp1* KO	Wild-type	*Parp1* KO
1	0.7	1.6	8.5	7.1
2	0.8	0.8	8.8	5.7
3	1.7	0.0	8.9	5.5
4	1.2	0.8	9.4	5.3
5	0.8	0.0	9.4	4.0
6	0.0	0.8	9.1	3.1
7	0.0	0.0	8.9	6.1
8	1.3	1.3	7.6	7.4
**mean**	**0.8**	**0.7**	**8.8**	**5.5**
**SD**	**0.6**	**0.6**	**0.6**	**1.5**
**SEM**	**0.2**	**0.2**	**0.2**	**0.5**
vs. WT		0.748		<0.001
vs. control			<0.001	<0.001

Normality Test (Shapiro-Wilk) : Passed (p = 0.289)

Equal Variance Test (Brown-Forsythe) : Passed (p = 0.091)

Two Way ANOVA

Source of Variation	DF	SS	MS	*F*	*p*
Cisplatin	1	332.262	332.262	417.038	<0.001
Parp1 KO	1	23.670	23.670	29.709	<0.001
Cisplatin x KO	1	19.845	19.845	24.909	<0.001
Residual	28	22.308	0.797		
Total	31	398.085	12.841		

Il1b EXPRESSION (DAY 1)

Fold vs. control in WT

Kidney	Control		Cisplatin	
no.	WT	*Parp1* KO	WT	*Parp1* KO
1	1.0	1.4	2.4	1.2
2	1.5	0.8	2.0	1.3
3	0.8	1.7	3.3	0.8
4	1.1	1.5	1.5	1.5
5	0.8	1.2	1.3	1.1
6	0.7	1.9	2.9	0.9
7	1.5	0.7	2.1	1.2
8	0.7	0.8	2.9	1.1
**mean**	**1.0**	**1.2**	**2.3**	**1.1**
**SD**	**0.3**	**0.4**	**0.7**	**0.2**
**SEM**	**0.1**	**0.2**	**0.3**	**0.1**

Logarithmic transformation

Kidney	Control		Cisplatin	
no.	WT	*Parp1* KO	WT	*Parp1* KO
1	-0.02	0.14	0.38	0.09
2	0.17	-0.12	0.29	0.10
3	-0.12	0.23	0.52	-0.11
4	0.04	0.17	0.17	0.17
5	-0.10	0.06	0.12	0.04
6	-0.16	0.27	0.46	-0.03
7	0.18	-0.13	0.33	0.07
8	-0.15	-0.08	0.46	0.05
**mean**	**-0.02**	**0.07**	**0.34**	**0.05**
**SD**	**0.14**	**0.16**	**0.14**	**0.09**
**SEM**	**0.05**	**0.06**	**0.05**	**0.03**
vs. WT		0.203		<0.001
vs. control			<0.001	0.765

Normality Test (Shapiro-Wilk) : Passed (p = 0.200)

Equal Variance Test (Brown-Forsythe) : Passed (p = 0.230)

Two Way ANOVA

Source of Variation	DF	SS	MS	*F*	*p*
Cisplatin	1	0.230	0.230	12.572	0.001
Parp1 KO	1	0.0830	0.0830	4.534	0.042
Cisplatin x KO	1	0.289	0.289	15.777	<0.001
Residual	28	0.512	0.0183		
Total	31	1.114	0.0359		

Il1b EXPRESSION (DAY 4)

Fold vs. control in WT

Kidney	Control		Cisplatin	
no.	WT	*Parp1* KO	WT	*Parp1* KO
1	0.9	1.6	62.4	4.7
2	1.5	0.8	50.2	6.7
3	0.7	1.8	54.9	14.8
4	1.1	1.7	67.2	6.7
5	0.8	1.3	45.3	6.9
6	0.7	2.2	39.3	9.2
7	1.6	0.8	33.0	13.0
8	0.7	0.9	32.1	15.8
**mean**	**1.0**	**1.4**	**48.0**	**9.7**
**SD**	**0.4**	**0.5**	**13.0**	**4.2**
**SEM**	**0.1**	**0.2**	**4.6**	**1.5**

Logarithmic transformation

Kidney	Control		Cisplatin	
no.	WT	*Parp1* KO	WT	*Parp1* KO
1	-0.03	0.20	1.80	0.67
2	0.18	-0.10	1.70	0.82
3	-0.14	0.26	1.74	1.17
4	0.02	0.22	1.83	0.83
5	-0.08	0.12	1.66	0.84
6	-0.15	0.33	1.59	0.96
7	0.20	-0.11	1.52	1.11
8	-0.17	-0.06	1.51	1.20
**mean**	**-0.02**	**0.11**	**1.67**	**0.95**
**SD**	**0.14**	**0.18**	**0.12**	**0.19**
**SEM**	**0.05**	**0.06**	**0.04**	**0.07**
vs. WT		0.111		<0.001
vs. control			<0.001	<0.001

Normality Test (Shapiro-Wilk) : Passed (p = 0.121)

Equal Variance Test (Brown-Forsythe) : Passed (p = 0.480)

Two Way ANOVA

Source of Variation	DF	SS	MS	*F*	*p*
Cisplatin	1	12.796	12.796	496.933	<0.001
Parp1 KO	1	0.684	0.684	26.561	<0.001
Cisplatin x KO	1	1.440	1.440	55.922	<0.001
Residual	28	0.721	0.0257		
Total	31	15.641	0.505		

Il6 EXPRESSION (DAY 1)

Fold vs. control in WT

Kidney	Control		Cisplatin	
no.	WT	*Parp1* KO	WT	*Parp1* KO
1	1.0	1.4	12.0	11.5
2	1.5	0.8	8.8	10.0
3	0.8	1.7	11.7	11.4
4	1.1	1.5	7.3	6.7
5	0.8	1.2	4.0	6.8
6	0.7	1.9	6.2	6.7
7	1.5	0.7	5.0	5.9
8	0.7	0.8	3.7	5.5
**mean**	**1.0**	**1.2**	**7.3**	**8.1**
**SD**	**0.3**	**0.4**	**3.3**	**2.5**
**SEM**	**0.1**	**0.2**	**1.2**	**0.9**

Logarithmic transformation

Kidney	Control		Cisplatin	
no.	WT	*Parp1* KO	WT	*Parp1* KO
1	-0.02	0.14	1.08	1.06
2	0.17	-0.12	0.95	1.00
3	-0.12	0.23	1.07	1.06
4	0.04	0.16	0.87	0.82
5	-0.10	0.06	0.60	0.83
6	-0.16	0.28	0.79	0.83
7	0.18	-0.13	0.70	0.77
8	-0.14	-0.08	0.56	0.74
**mean**	**-0.02**	**0.07**	**0.83**	**0.89**
**SD**	**0.14**	**0.16**	**0.20**	**0.13**
**SEM**	**0.05**	**0.06**	**0.07**	**0.05**
vs. WT		0.279		0.438
vs. control			<0.001	<0.001

Normality Test (Shapiro-Wilk) : Passed (p = 0.138)

Equal Variance Test (Brown-Forsythe) : Passed (p = 0.485)

Two Way ANOVA

Source of Variation	DF	SS	MS	*F*	*p*
Cisplatin	1	5.557	5.557	219.015	<0.001
Parp1 KO	1	0.0453	0.0453	1.785	0.192
Cisplatin x KO	1	0.00127	0.00127	0.0502	0.824
Residual	28	0.710	0.0254		
Total	31	6.314	0.204		

Il6 EXPRESSION (DAY 4)

Fold vs. control in WT

Kidney	Control		Cisplatin	
no.	WT	*Parp1* KO	WT	*Parp1* KO
1	0.7	0.7	20.5	8.8
2	0.8	0.6	28.4	9.0
3	1.5	1.3	24.6	3.5
4	1.0	0.5	13.7	5.8
5	0.8	0.3	25.5	21.9
6	0.8	1.4	21.0	16.4
7	0.9	1.1	37.2	18.9
8	1.5	1.2	40.8	17.4
**mean**	**1.0**	**0.9**	**26.5**	**12.7**
**SD**	**0.3**	**0.4**	**8.9**	**6.8**
**SEM**	**0.1**	**0.1**	**3.1**	**2.4**

Logarithmic transformation

Kidney	Control		Cisplatin	
no.	WT	*Parp1* KO	WT	*Parp1* KO
1	-0.14	-0.17	1.31	0.94
2	-0.09	-0.21	1.45	0.95
3	0.17	0.12	1.39	0.55
4	0.00	-0.30	1.14	0.76
5	-0.09	-0.49	1.41	1.34
6	-0.10	0.15	1.32	1.21
7	-0.05	0.06	1.57	1.28
8	0.17	0.07	1.61	1.24
**mean**	**-0.02**	**-0.10**	**1.40**	**1.04**
**SD**	**0.12**	**0.23**	**0.15**	**0.28**
**SEM**	**0.04**	**0.08**	**0.05**	**0.10**
vs. WT		0.418		0.005
vs. control			<0.001	<0.001

Normality Test (Shapiro-Wilk) : Passed (p = 0.071)

Equal Variance Test (Brown-Forsythe) : Failed (p < 0.050)

Two-factor linear model with HC3 robust SE (Type III Wald *F*)

Source of Variation	DF	Wald *F*	*p*
Cisplatin	1, 28	267.196	<0.001
Parp1 KO	1, 28	8.207	0.004
Cisplatin x KO	1, 28	3.377	0.066

Ccl5 EXPRESSION (DAY 1)

Fold vs. control in WT

Kidney	Control		Cisplatin	
no.	WT	*Parp1* KO	WT	*Parp1* KO
1	0.9	1.3	1.4	1.4
2	1.2	1.1	1.0	1.2
3	0.8	0.8	1.2	1.2
4	0.7	1.0	0.8	1.6
5	1.5	1.4	1.1	1.0
6	1.1	1.1	1.0	1.4
7	1.1	1.0	0.7	1.2
8	0.7	1.1	1.0	1.2
**mean**	**1.0**	**1.1**	**1.1**	**1.3**
**SD**	**0.3**	**0.2**	**0.2**	**0.2**
**SEM**	**0.1**	**0.1**	**0.1**	**0.1**
vs. WT		0.339		0.059
vs. control			0.645	0.155

Normality Test (Shapiro-Wilk) : Passed (p = 0.321)

Equal Variance Test (Brown-Forsythe) : Passed (p = 0.698)

Two Way ANOVA

Source of Variation	DF	SS	MS	*F*	*p*
Cisplatin	1	0.0915	0.0915	1.856	0.184
Parp1 KO	1	0.214	0.214	4.329	0.047
Cisplatin x KO	1	0.0245	0.0245	0.496	0.487
Residual	28	1.381	0.0493		
Total	31	1.711	0.0552		

Ccl5 EXPRESSION (DAY 4)

Fold vs. control in WT

Kidney	Control		Cisplatin	
no.	WT	*Parp1* KO	WT	*Parp1* KO
1	1.0	0.9	35.8	4.4
2	1.0	0.3	17.9	7.0
3	1.0	1.5	14.4	8.3
4	0.7	0.4	28.2	13.6
5	1.3	0.4	33.0	13.9
6	1.1	0.4	26.6	16.6
7	1.2	1.0	25.3	8.5
8	0.8	1.1	31.5	7.7
**mean**	**1.0**	**0.8**	**26.6**	**10.0**
**SD**	**0.2**	**0.4**	**7.3**	**4.2**
**SEM**	**0.1**	**0.2**	**2.6**	**1.5**

Logarithmic transformation

Kidney	Control		Cisplatin	
no.	WT	*Parp1* KO	WT	*Parp1* KO
1	-0.01	-0.04	1.55	0.65
2	0.01	-0.59	1.25	0.85
3	0.01	0.17	1.16	0.92
4	-0.16	-0.43	1.45	1.13
5	0.10	-0.37	1.52	1.14
6	0.03	-0.35	1.43	1.22
7	0.08	0.01	1.40	0.93
8	-0.12	0.05	1.50	0.89
**mean**	**-0.01**	**-0.19**	**1.41**	**0.97**
**SD**	**0.09**	**0.27**	**0.14**	**0.19**
**SEM**	**0.03**	**0.10**	**0.05**	**0.07**
vs. WT		0.099		<0.001
vs. control			<0.001	<0.001

Normality Test (Shapiro-Wilk) : Passed (p = 0.989)

Equal Variance Test (Brown-Forsythe) : Failed (p < 0.050)

Two-factor linear model with HC3 robust SE (Type III Wald *F*)

Source of Variation	DF	Wald *F*	*p*
Cisplatin	1, 28	339.685	<0.001
Parp1 KO	1, 28	20.172	<0.001
Cisplatin x KO	1, 28	3.331	0.079

Cxcl1 EXPRESSION (DAY 1)

Fold vs. control in WT

Kidney no.	Control WT	Control *Parp1* KO	Cisplatin WT	Cisplatin *Parp1* KO
1	1.0	1.4	47.9	32.3
2	1.2	1.2	48.7	23.7
3	0.8	0.8	41.9	27.9
4	0.7	1.0	44.3	24.5
5	1.5	1.4	56.1	31.8
6	1.1	1.1	33.1	27.3
7	1.1	1.0	57.9	23.6
8	0.7	1.1	30.6	23.8
**mean**	**1.0**	**1.1**	**45.1**	**26.9**
**SD**	**0.3**	**0.2**	**9.8**	**3.6**
**SEM**	**0.1**	**0.1**	**3.5**	**1.3**

Logarithmic transformation

Kidney no.	Control WT	Control *Parp1* KO	Cisplatin WT	Cisplatin *Parp1* KO
1	-0.02	0.14	1.68	1.51
2	0.07	0.07	1.69	1.38
3	-0.10	-0.09	1.62	1.45
4	-0.15	-0.01	1.65	1.39
5	0.18	0.16	1.75	1.50
6	0.02	0.03	1.52	1.44
7	0.04	0.02	1.76	1.37
8	-0.16	0.05	1.49	1.38
**mean**	**-0.01**	**0.05**	**1.64**	**1.43**
**SD**	**0.12**	**0.08**	**0.10**	**0.06**
**SEM**	**0.04**	**0.03**	**0.04**	**0.02**
vs. WT		0.201		<0.001
vs. control			<0.001	<0.001

Normality Test (Shapiro-Wilk) : Passed (p = 0.752)
Equal Variance Test (Brown-Forsythe) : Passed (p = 0.381)
Two Way ANOVA

Source of Variation	DF	SS	MS	*F*	*p*
Cisplatin	1	18.465	18.465	2228.825	<0.001
Parp1 KO	1	0.0503	0.0503	6.076	0.020
Cisplatin x KO	1	0.154	0.154	18.638	<0.001
Residual	28	0.232	0.00828		
Total	31	18.902	0.610		

Cxcl1 EXPRESSION (DAY 4)

Fold vs. control in WT

Kidney no.	Control WT	Control *Parp1* KO	Cisplatin WT	Cisplatin *Parp1* KO
1	0.9	1.3	109.9	68.0
2	1.2	1.0	126.7	45.5
3	0.8	0.7	160.9	55.1
4	0.7	0.9	254.6	68.6
5	1.6	1.3	154.5	122.9
6	1.0	1.0	254.6	99.6
7	1.1	1.0	218.5	113.3
8	0.7	1.0	217.8	122.3
**mean**	**1.0**	**1.0**	**187.2**	**86.9**
**SD**	**0.3**	**0.2**	**56.6**	**31.2**
**SEM**	**0.1**	**0.1**	**20.0**	**11.0**

Logarithmic transformation

Kidney no.	Control WT	Control *Parp1* KO	Cisplatin WT	Cisplatin *Parp1* KO
1	-0.03	0.11	2.04	1.83
2	0.09	0.01	2.10	1.66
3	-0.11	-0.15	2.21	1.74
4	-0.16	-0.07	2.41	1.84
5	0.20	0.10	2.19	2.09
6	0.01	0.00	2.41	2.00
7	0.03	-0.02	2.34	2.05
8	-0.17	0.00	2.34	2.09
**mean**	**-0.02**	**0.00**	**2.25**	**1.91**
**SD**	**0.13**	**0.08**	**0.14**	**0.17**
**SEM**	**0.05**	**0.03**	**0.05**	**0.06**
vs. WT		0.111		<0.001
vs. control			<0.001	<0.001

Normality Test (Shapiro-Wilk) : Passed (p = 0.512)
Equal Variance Test (Brown-Forsythe) : Passed (p = 0.064)
Two Way ANOVA

Source of Variation	DF	SS	MS	*F*	*p*
Cisplatin	1	35.024	35.024	1978.253	<0.001
Parp1 KO	1	0.213	0.213	12.034	0.002
Cisplatin x KO	1	0.254	0.254	14.360	<0.001
Residual	28	0.496	0.0177		
Total	31	35.987	1.161		

Cxcl2 EXPRESSION (DAY 1)

Fold vs. control in WT

Kidney no.	Control WT	Control *Parp1* KO	Cisplatin WT	Cisplatin *Parp1* KO
1	1.1	0.6	62.8	59.3
2	1.0	0.5	64.1	42.1
3	1.2	0.7	77.3	62.3
4	1.1	0.6	67.4	46.9
5	0.8	0.9	72.3	48.4
6	0.9	1.2	59.5	52.7
7	1.1	1.2	69.1	66.3
8	0.8	0.8	72.8	38.0
**mean**	**1.0**	**0.8**	**68.2**	**52.0**
**SD**	**0.1**	**0.3**	**5.9**	**10.0**
**SEM**	**0.1**	**0.1**	**2.1**	**3.5**

Logarithmic transformation

Kidney no.	Control WT	Control *Parp1* KO	Cisplatin WT	Cisplatin *Parp1* KO
1	0.05	-0.22	1.80	1.77
2	0.00	-0.29	1.81	1.62
3	0.08	-0.14	1.89	1.79
4	0.03	-0.20	1.83	1.67
5	-0.11	-0.03	1.86	1.69
6	-0.04	0.06	1.77	1.72
7	0.03	0.08	1.84	1.82
8	-0.07	-0.07	1.86	1.58
**mean**	**0.00**	**-0.10**	**1.83**	**1.71**
**SD**	**0.07**	**0.14**	**0.04**	**0.08**
**SEM**	**0.02**	**0.05**	**0.01**	**0.03**
vs. WT		0.094		0.003
vs. control			<0.001	<0.001

Normality Test (Shapiro-Wilk) : Passed (p = 0.985)
Equal Variance Test (Brown-Forsythe) : Failed (p < 0.050)
Two-factor linear model with HC3 robust SE (Type III Wald *F*)

Source of Variation	DF	Wald *F*	*p*
Cisplatin	1, 28	915.85	<0.001
Parp1 KO	1, 28	12.87	0.001
Cisplatin x KO	1, 28	0.17	0.681

Cxcl2 EXPRESSION (DAY 4)

Fold vs. control in WT

Kidney no.	Control WT	Control *Parp1* KO	Cisplatin WT	Cisplatin *Parp1* KO
1	0.7	1.4	106.4	48.8
2	0.6	1.2	193.7	77.0
3	0.9	1.4	119.9	54.6
4	0.7	1.3	109.5	52.1
5	1.2	1.0	145.2	54.9
6	1.5	1.1	184.4	53.7
7	1.4	1.3	165.5	72.4
8	1.1	1.0	172.9	86.7
**mean**	**1.0**	**1.2**	**149.7**	**62.5**
**SD**	**0.3**	**0.2**	**34.5**	**14.1**
**SEM**	**0.1**	**0.1**	**12.2**	**5.0**

Logarithmic transformation

Kidney no.	Control WT	Control *Parp1* KO	Cisplatin WT	Cisplatin *Parp1* KO
1	-0.16	0.15	2.03	1.69
2	-0.23	0.07	2.29	1.89
3	-0.04	0.15	2.08	1.74
4	-0.14	0.12	2.04	1.72
5	0.06	-0.02	2.16	1.74
6	0.16	0.03	2.27	1.73
7	0.15	0.12	2.22	1.86
8	0.02	0.00	2.24	1.94
**mean**	**-0.02**	**0.08**	**2.16**	**1.79**
**SD**	**0.15**	**0.07**	**0.10**	**0.09**
**SEM**	**0.05**	**0.02**	**0.04**	**0.03**
vs. WT		0.077		<0.001
vs. control			<0.001	<0.001

Normality Test (Shapiro-Wilk) : Passed (p = 0.524)
Equal Variance Test (Brown-Forsythe) : Passed (p = 0.186)
Two Way ANOVA

Source of Variation	DF	SS	MS	*F*	*p*
Cisplatin	1	30.367	30.367	2631.614	<0.001
Parp1 KO	1	0.156	0.156	13.492	0.001
Cisplatin x KO	1	0.453	0.453	39.300	<0.001
Residual	28	0.323	0.0115		
Total	31	31.299	1.010		

p-p65 EXPRESSION (DAY 4)

Fold vs. control in WT

Kidney no.	Control WT	Control *Parp1* KO	Cisplatin WT	Cisplatin *Parp1* KO
1	1.01	1.15	1.38	1.01
2	1.15	1.09	1.58	1.13
3	1.06	0.97	1.50	0.98
4	1.01	1.07	1.53	1.02
5	0.91	1.07	1.47	1.04
6	0.86	0.97	1.52	1.31
**mean**	**1.00**	**1.05**	**1.49**	**1.08**
**SD**	**0.10**	**0.07**	**0.07**	**0.12**
**SEM**	**0.04**	**0.03**	**0.03**	**0.05**
vs. WT		0.337		<0.001
vs. control			<0.001	0.607

Normality Test (Shapiro-Wilk) : Passed (p = 0.407)
Equal Variance Test (Brown-Forsythe) : Passed (p = 0.829)
Two Way ANOVA

Source of Variation	DF	SS	MS	*F*	*p*
Cisplatin	1	0.410	0.410	46.332	<0.001
Parp1 KO	1	0.193	0.193	21.854	<0.001
Cisplatin x KO	1	0.326	0.326	36.813	<0.001
Residual	20	0.177	0.00885		
Total	23	1.106	0.0481		

t-p65 EXPRESSION (DAY 4)

Fold vs. control in WT

Kidney no.	Control WT	Control *Parp1* KO	Cisplatin WT	Cisplatin *Parp1* KO
1	0.90	1.08	0.69	0.53
2	1.08	1.10	0.72	0.81
3	1.00	0.94	0.74	0.53
4	0.95	1.08	0.68	0.77
5	1.03	1.03	0.75	0.80
6	1.04	0.94	0.70	0.81
**mean**	**1.00**	**1.03**	**0.72**	**0.71**
**SD**	**0.07**	**0.07**	**0.03**	**0.14**
**SEM**	**0.03**	**0.03**	**0.01**	**0.06**
vs. WT		0.598		0.892
vs. control			<0.001	<0.001

Normality Test (Shapiro-Wilk) : Passed (p = 0.056)
Equal Variance Test (Brown-Forsythe) : Passed (p = 0.340)
Two Way ANOVA

Source of Variation	DF	SS	MS	*F*	*p*
Cisplatin	1	0.542	0.542	74.669	<0.001
Parp1 KO	1	0.000577	0.000577	0.0795	0.781
Cisplatin x KO	1	0.00165	0.00165	0.227	0.639
Residual	20	0.145	0.00726		
Total	23	0.690	0.0300		

p65 ACTIVATION (p-p65/t-p65 ratio) (DAY 4)

Fold vs. control in WT

Kidney no.	Control WT	Control *Parp1* KO	Cisplatin WT	Cisplatin *Parp1* KO
1	1.12	1.07	1.99	1.89
2	1.07	1.00	2.18	1.40
3	1.05	1.03	2.02	1.84
4	1.06	0.99	2.23	1.33
5	0.89	1.04	1.95	1.30
6	0.83	1.03	2.17	1.61
**mean**	**1.00**	**1.03**	**2.09**	**1.56**
**SD**	**0.12**	**0.03**	**0.12**	**0.26**
**SEM**	**0.05**	**0.01**	**0.05**	**0.11**
vs. WT		0.665		<0.001
vs. control			<0.001	<0.001

Normality Test (Shapiro-Wilk) : Passed (p = 0.702)
Equal Variance Test (Brown-Forsythe) : Failed (p < 0.050)
Two-factor linear model with HC3 robust SE (Type III Wald *F*)

Source of Variation	DF	Wald *F*	*p*
Cisplatin	1, 20	21.045	<0.001
Parp1 KO	1, 20	17.253	<0.001
Cisplatin x KO	1, 20	16.019	<0.001

COX-2 EXPRESSION (DAY 4)

Fold vs. control in WT

Kidney no.	Control WT	Control *Parp1* KO	Cisplatin WT	Cisplatin *Parp1* KO
1	1.03	1.05	1.39	1.06
2	0.83	0.86	1.59	1.24
3	1.15	1.07	1.31	0.99
4	1.02	1.05	1.34	1.11
5	0.74	0.80	1.76	1.22
6	1.23	1.03	1.18	1.08
**mean**	**1.00**	**0.98**	**1.43**	**1.12**
**SD**	**0.19**	**0.12**	**0.21**	**0.10**
**SEM**	**0.08**	**0.05**	**0.09**	**0.04**
vs. WT		0.795		0.003
vs. control			<0.001	0.139

Normality Test (Shapiro-Wilk) : Passed (p = 0.971)
Equal Variance Test (Brown-Forsythe) : Passed (p = 0.519)
Two Way ANOVA

Source of Variation	DF	SS	MS	*F*	*p*
Cisplatin	1	0.485	0.485	19.063	<0.001
Parp1 KO	1	0.167	0.167	6.562	0.019
Cisplatin x KO	1	0.122	0.122	4.792	0.041
Residual	20	0.509	0.0254		
Total	23	1.283	0.0558		

Ptgs2 EXPRESSION (DAY 1)

Fold vs. control in WT

Kidney no.	Control WT	Control *Parp1* KO	Cisplatin WT	Cisplatin *Parp1* KO
1	0.8	1.6	22.2	21.1
2	1.2	1.0	21.0	19.3
3	0.6	1.7	15.8	17.3
4	0.9	0.7	16.0	22.9
5	1.4	1.2	18.1	14.8
6	1.3	0.8	7.9	21.3
7	0.7	0.5	14.0	22.9
8	1.1	1.3	8.9	19.8
**mean**	**1.0**	**1.1**	**15.5**	**19.9**
**SD**	**0.3**	**0.4**	**5.2**	**2.8**
**SEM**	**0.1**	**0.1**	**1.8**	**1.0**

Logarithmic transformation

Kidney no.	Control WT	Control *Parp1* KO	Cisplatin WT	Cisplatin *Parp1* KO
1	-0.10	0.20	1.35	1.32
2	0.08	0.02	1.32	1.29
3	-0.22	0.22	1.20	1.24
4	-0.05	-0.13	1.20	1.36
5	0.15	0.06	1.26	1.17
6	0.11	-0.08	0.90	1.33
7	-0.16	-0.28	1.15	1.36
8	0.04	0.10	0.95	1.30
**mean**	**-0.02**	**0.01**	**1.17**	**1.30**
**SD**	**0.13**	**0.17**	**0.16**	**0.06**
**SEM**	**0.05**	**0.06**	**0.06**	**0.02**
vs. WT		0.655		0.072
vs. control			<0.001	<0.001

Normality Test (Shapiro-Wilk) : Passed (p = 0.264)
Equal Variance Test (Brown-Forsythe) : Passed (p = 0.212)
Two Way ANOVA

Source of Variation	DF	SS	MS	*F*	*p*
Cisplatin	1	12.135	12.135	623.870	<0.001
Parp1 KO	1	0.0523	0.0523	2.688	0.112
Cisplatin x KO	1	0.0195	0.0195	1.002	0.325
Residual	28	0.545	0.0195		
Total	31	12.752	0.411		

Ptgs2 EXPRESSION (DAY 4)

Fold vs. control in WT

Kidney no.	Control WT	Control *Parp1* KO	Cisplatin WT	Cisplatin *Parp1* KO
1	0.7	1.6	112.8	50.5
2	1.3	1.1	205.3	74.9
3	0.7	1.7	127.1	40.1
4	0.7	0.6	116.0	60.5
5	1.5	1.0	153.9	57.4
6	1.5	0.6	195.4	62.1
7	0.6	0.6	175.4	60.8
8	1.0	1.1	183.2	47.4
**mean**	**1.0**	**1.0**	**158.6**	**56.7**
**SD**	**0.4**	**0.4**	**36.5**	**10.6**
**SEM**	**0.1**	**0.2**	**12.9**	**3.8**

Logarithmic transformation

Kidney no.	Control WT	Control *Parp1* KO	Cisplatin WT	Cisplatin *Parp1* KO
1	-0.16	0.22	2.05	1.70
2	0.13	0.03	2.31	1.87
3	-0.14	0.23	2.10	1.60
4	-0.17	-0.23	2.06	1.78
5	0.18	0.00	2.19	1.76
6	0.17	-0.20	2.29	1.79
7	-0.25	-0.20	2.24	1.78
8	-0.01	0.03	2.26	1.68
**mean**	**-0.03**	**-0.02**	**2.19**	**1.75**
**SD**	**0.17**	**0.18**	**0.10**	**0.08**
**SEM**	**0.06**	**0.06**	**0.04**	**0.03**
vs. WT		0.861		<0.001
vs. control			<0.001	<0.001

Normality Test (Shapiro-Wilk) : Passed (p = 0.224)
Equal Variance Test (Brown-Forsythe) : Passed (p = 0.084)
Two Way ANOVA

Source of Variation	DF	SS	MS	*F*	*p*
Cisplatin	1	31.745	31.745	1575.089	<0.001
Parp1 KO	1	0.371	0.371	18.384	0.001
Cisplatin x KO	1	0.415	0.415	20.591	<0.001
Residual	28	0.564	0.0202		
Total	31	33.095	1.068		

Kidney PGE2 (DAY 4)

pg/mg protein

Kidney no.	Control		Cisplatin	
	WT	*Parp1* KO	WT	*Parp1* KO
1	21.6	31.1	74.7	38.3
2	20.5	23.9	60.2	23.1
3	29.0	23.0	60.9	34.9
4	30.8	25.9	75.4	16.6
5	24.8	30.6	72.0	31.9
6	28.6	27.8	80.9	33.1
7	23.5	30.3	51.0	20.9
8	22.0	26.6	83.9	35.5
**mean**	**25.1**	**27.4**	**69.9**	**29.3**
**SD**	**3.9**	**3.1**	**11.4**	**8.0**
**SEM**	**1.4**	**1.1**	**4.0**	**2.8**
vs. WT		0.541		<0.001
vs. control			<0.001	0.615

Normality Test (Shapiro-Wilk) : Passed (p = 0.596)

Equal Variance Test (Brown-Forsythe) : Passed (p = 0.051)

Two Way ANOVA

Source of Variation	DF	SS	MS	*F*	*p*
Cisplatin	1	4351.244	4351.244	79.924	<0.001
Parp1 KO	1	2936.857	2936.857	53.945	<0.001
Cisplatin x KO	1	3678.330	3678.330	67.564	<0.001
Residual	28	1524.375	54.442		
Total	31	12490.807	402.929		
